# Changing patterns in marriage choice and related health risk in the Pakistani heritage community in Bradford UK: a qualitative study.

**DOI:** 10.12688/wellcomeopenres.23338.2

**Published:** 2025-03-27

**Authors:** Neil Small, Rifat Razaq, Vishal Sharma, Alice Cunningham, Zuneera Khurshid, Shahid Islam

**Affiliations:** 1 University of Bradford Faculty of Health Studies, Bradford, UK; 2 Improvement Academy, Bradford Institute for Health Research, Bradford, UK; 3 Bradford Institute for Health Research, Bradford, UK

**Keywords:** Consanguinity: childhood morbidity: Pakistani heritage: focus groups

## Abstract

**Background:**

Children born to blood relations (consanguineous parents), primarily cousins, have higher mortality and morbidity than children born to non-consanguineous parents. Consanguinity is low in the UK but high in some communities, including the Pakistani heritage community in Bradford. There has been a marked decrease in consanguinity in the last decade and that is likely to result in reductions in excess mortality and morbidity.

**Methods:**

Drawing on patterns of child health reported by the Born in Bradford study, augmented with a summary of the literature on motivations for choosing consanguineous unions and on the shifting characteristics of those who make this choice, questions about marriage choice and knowledge of allied health risk were devised. They were explored in four focus groups with self-identified members of the Pakistani heritage community. Groups were divided by age and gender. Discussions were analysed using Thematic Analysis.

**Results:**

There was agreement that rates of consanguinity were declining. Older group members were concerned this might indicate a shift away from tradition and damage community cohesion. Younger participants were positive about the benefits of a shift towards individual choice. They felt this could be achieved without damaging community strengths. Reasons for the fall in numbers were attributed to changes within the community, including higher numbers of people staying in education beyond school. External factors, including new immigration rules, were also considered.

There was not a consensus about health risks, some older respondents were sceptical of links between marriage choice and child health and concerned about how health risks were communicated. All were concerned that marriage choice should not be used to demonise this community.

**Conclusion:**

A commitment to sustaining community cohesion is shared by all groups. Younger people think this can be achieved despite falls in consanguinity. There are continuing challenges in communicating health risk.

## Plain Language Summary

The “Born in Bradford” (BiB) study into children’s health and well-being started in 2007. BiB researchers found that children whose parents are blood relations, typically cousins, are more likely to have ill-health than children whose parents are not related. Marriages between blood relations are rare in the UK but are common in those of Pakistani heritage in Bradford, although there has been a decrease in these marriages in recent years. Working with Bradford Council, BiB researchers undertook group discussions with members of the Pakistani community to understand why these sorts of marriages have decreased and to explore understandings about health risks for children born to parents who are related. Discussions highlighted the importance of people staying longer in education, social change encouraging individual rather than family decision making and external factors including changes in immigration laws. There was a concern that linking worse child health with cousin marriage was too simplistic and an allied concern about how health risks are communicated.

This is a small scale study in one city but it adds to wider literature seeking to understand health implications of marriage choice. Around one billion people across the world live in countries where marrying a cousin is common.

## Background

### The setting

Bradford is a city in the north of England with a population of around a half-million. Sixty percent of babies born in the city are born into the poorest 20% of the population of England and Wales. The city is the site of two ongoing birth cohort studies, Born in Bradford (BiB) recruited 12453 women who had 13776 pregnancies between 2007 and 2011. In addition, 3448 of these women’s partners were also recruited (see Raynor and the Born in Bradford Collaborative Group, 2008 for the study protocol and Wright *et al.*, 2013 to see cohort characteristics). A second cohort study, Born in Bradford’s Better Start (BiBBS), selected from three inner city wards within the wider catchment area of BiB, recruited 2392 mothers who had 2626 pregnancies between 2016 and 2019 ( Dickerson *et al.*, 2016; Dickerson *et al.*, 2022). The health, wellbeing and educational attainment of the children born to BiB and BiBBS mothers has been followed up from their birth.

In both cohorts the majority of women were of Pakistani heritage, 65% and 62% in BiB and BiBBS respectively. These percentages were close to the profile of all births in the city in the study recruitment years. The majority, 64%, of Pakistani-heritage mothers recruited to Born in Bradford between 2007 and 2010 were related by blood to the father of their child. These are termed consanguineous unions and are generally categorised as either first cousin unions or “other blood”, most often second cousins. First cousins share a common grandparent and second cousins share a common great-grandparent. By the time of recruitment to BiBBS rates of consanguinity had fallen and 46% of Pakistani heritage mothers reported they were in consanguineous unions. All but one White British respondent was unrelated to their baby’s father in both cohorts, and around 90% of the ‘Other ethnicities’ group (i.e., not White British or Pakistani heritage) were unrelated to the baby’s father in both cohorts.

The qualitative study we report seeks to understand the contemporary practice of consanguinity in Bradfordians of Pakistani heritage. It is prompted by two recent quantitative studies. The first, introduced above, identified a significant reduction in rates of consanguinity between mothers recruited to BiB and those recruited around nine years later to BiBBS, and it examined the changing characteristics of the consanguineous. The reduction in consanguinity was most marked in women of Pakistani heritage who were born in the UK, in those educated to A level or higher and in women under age 25 ( Small *et al.*, 2024a). The second study looked at mortality, morbidity and educational outcomes in BiB children up to age 10. It identified marked disadvantages for children of consanguineous unions ( Small *et al.*, 2024b). Our qualitative study seeks to:

- Consider if the reduction in consanguinity we have reported, and the changing characteristics of the consanguineous, is recognised by Bradfordians of Pakistani origin; to ask what they attribute the change to and to discuss what impact they think it has on their community.
- Examine to what extent health and education outcomes linked to consanguinity are known in the Pakistani heritage community, to assess the significance they are given and to seek advice about how such outcomes can best be discussed.

### What consanguinity is and why we are interested in it?

Around one billion people, one in eight of the world’s population, live in countries where marriage among relatives is common (classified as where such marriages constitute above 20% of all marriages – see Bittles, 2012). Consanguinity is very low in the UK although it is common in some communities, including those of Pakistani heritage.

Worldwide, the published literature indicating an increase in infant and childhood morbidity in the children of consanguineous couples is extensive, as is a recognition of deaths in infancy being higher in children of first cousin unions when compared with non-consanguineous couples ( Bittles, 2012, 136). Amongst this extensive literature there have been estimates of the impact of cousin marriage on health and on socioeconomics in South Asia ( Mobarak *et al.*, 2019) and on both rates of consanguinity and under five mortality and morbidity across Pakistan, including how these have changed over time ( Bashir & Nazir, 2022; Mustafa *et al.*, 2017). Studies in Pakistan focussed on regions and specific localities have looked at the characteristics of the consanguineous ( Ahmad *et al.*, 2016; Hina & Malik, 2015; Riaz *et al.*, 2016; Shenk *et al.*, 2024). In addition, there have been studies and reviews of perceptions about consanguinity, including in Pakistan ( Sarkar, 2024), Qatar ( Abdu *et al.*, 2023) and South India ( Joseph *et al.*, 2014).

In 2013 BiB research reported that the frequency of recessive genetic disorders among children born to consanguineous parents is around twice that of children of non-related parents ( Sheridan *et al.*, 2013). BiB’s ongoing studies also reported higher levels of mortality and morbidity in BiB children whose parents are first cousins compared with children whose parents are not related. Morbidity was ascertained by counts of primary care appointments and prescriptions and by numbers of accident and emergency and hospital outpatient attendances. There are also differences between children born to first cousins and those whose parents are not related in rates of speech and language development difficulties and learning difficulties. (The same sort of differences are evident in children born to parents who are second cousins, but they are less pronounced.) Differences are also evident in a series of measures related to the child’s education. Here children born to first cousins do less well in reaching phonics standards, and in reaching a good stage of development and there are higher numbers of children with special education needs ( Small *et al.*, 2024b). Born in Bradford has also identified some putative health benefits to BiB’s mothers associated with their being in consanguineous marriages via enhancing family stability and through the potential benefits of communal social capital ( Bhopal *et al.*, 2014).

### The challenges involved in discussing genetic risk and consanguinity

Congenital anomalies occur in all communities. When BiB researchers reported that consanguinity was associated with a doubling of risk for congenital anomalies that doubling still meant that nearly 94 children in every 100 born to consanguineous partners were born without an anomaly. About 97 in every 100 born to non-consanguineous parents did not have an anomaly. Congenital anomalies occurred in 6.2% of babies born to first cousins and in 2.6% of babies born to unrelated parents. When children are born to consanguineous unions there is an increased probability that some of the DNA a child inherits will be the same in copies they inherit from father and mother because it comes from a single common ancestor. When DNA is inherited in this way it is called autozygosity. Some DNA variants are recessive variants, they only have an impact on the child if they are present in both copies of the DNA. That impact can be manifest in congenital anomalies in the child. It is more likely that each parent will have the same variant if they are related by blood, but non-blood related parents can also have the same variant.

Patterns of marrying within population substructures, or within restricted population groups, result in substantial population stratification. This practice, termed endogamy, can increase the risk of congenital anomalies. In Bradford’s Pakistani heritage population biraderi is of particular interest. Biraderi are patrilineal affiliations associated with occupation, kinship group and place of origin. They embody shared understandings of social hierarchy and marrying within one’s biraderi has been seen as desirable. Sheridan *et al.* did not consider biraderi affiliation. Although participants were asked what their biraderi was, including this in the analysis was not possible because there were too many who replied that they did not know. Subsequent to the Sheridan *et al.* paper biraderi and endogamy has been explored in the BiB cohort (see Bittles & Small, 2016; Small *et al.*, 2017).

There are also other significant factors associated with increased presence of congenital anomalies. BiB reported an increase in risk of similar magnitude to consanguinity for non-consanguineous mothers of white British origin older than 34 years. Further, anomalies of different sorts and with risk factors other than consanguinity occur in all babies. In BiB 31% of all anomalies in children of Pakistani origin could be attributed to consanguinity ( Sheridan *et al.*, 2013). (See Bhinder *et al.*, 2019 about the potential for long-term population advantages of consanguinity).

The literature on the impact of consanguinity on health include debates about the significance of the variables taken into consideration when calculating risk. Although Mobarak *et al.* (2019) reported increases in foetal anomalies that were similar to Sheridan *et al.* (2013) their paper is critical of the way the calculation of the impact of consanguinity on mortality and morbidity is arrived at. They argue that the availability of unmarried cousins at the time a marriage is being planned creates quasi-random variation in the propensity to marry consanguineously. When this was taken into account in their study, and also when health outcomes across offspring from the same union were considered, the impact of consanguinity on mortality and morbidity was statistically insignificant. Sheridan *et al.* (2013) did not include a consideration of health outcomes across offspring from the same union. There were couples in the Born in Bradford study who had more than one child during the years the study recruited participants but their numbers were too small to allow meaningful analysis. We did not collect data on siblings of the children in Born in Bradford if they were born before or after our recruitment years. Nor did we know numbers of cousins available for marriage.

We have then a complex picture to paint – congenital anomalies are relatively rare, most children born to consanguineous parents will not have them. They occur in all communities irrespective of marriage type and the majority of anomalies, including in children of Pakistani origin parents, do not have consanguinity as a risk factor. Choosing a marriage partner from a restricted population group increases the risk of anomalies even if it is not apparent that both partners are related. There is also a debate about how figures linking cousin marriage and enhanced risk take account of possible confounding factors.

But, the consequences of being born with a congenital anomaly can be severe, infant mortality and long term morbidity, and given high numbers of births to consanguineous parents in Bradford, numbers dying or living with a congenital anomaly are significant. Congenital anomalies and other morbidities are a profound challenge for the individuals and families in which they occur and are a factor that health, social care and education providers should be conscious of when they plan service provision especially in areas where consanguinity is common.

This complex picture presents a challenge for public engagement and for health promotion and health education. That challenge is exacerbated by a public discourse that can be hostile not just to consanguinity but can also use consanguinity as an example of cultural practices that are singled out and blamed for illness and for generating increased health burden on public services. Some discussion engages with how to reduce genetic risk and this ranges from a “ban cousin marriage” rhetoric to a considered discussion as to why there is a propensity to have this sort of marriage. A consideration of the law and consanguinity ( Nash, 2024) cites examples of rhetorical, simplistic and stigmatising public discourse, a genre that not unreasonably can be seen as a rod used to beat an already stigmatised community.

## Why cousin marriage?

Consanguinity is an area of interest for many disciplines, these include genetics, epidemiology, medicine and health, theology, the law, anthropology and sociology. In this limited review we focus on those areas of interest that have been apparent in our previous work with the Pakistani heritage community in Bradford and which have links with the two studies, introduced above, that inform this qualitative study.

### Economic factors and length of time in education

Considerations as to why people marry cousins characteristically cite economic, cultural and religious motivations ( Bittles, 2012). Economic benefits have focussed on the protection of land and property so that these remain within the same extended family. This engages with survival needs in poor families and with the retention of wealth and the enhancement of socially advantageous kinship ties in wealthier families (see Shaw, 2014). For example, Mobarak *et al.* (2019) describes how the possibility of different dowry arrangement in Pakistan, specifically allowing deferral of dowry payments until after marriage, constitutes an economic benefit of consanguineous marriage.

The gap between study recruitment for BiB and BiBBS saw an economic recession and a period of austerity in public spending. Our data does not identify an adverse impact of recession and austerity on the population’s sense of economic wellbeing in the BiBBS sample. By 2016–2019 the Pakistani heritage population reported feeling more economically secure when compared with the BiB recruitment period of 2007–2010 ( Uphoff *et al.*, 2018). But these were small changes in felt economic security and were not of an order that suggest that changes in Bradford’s consanguinity rates are shaped by any change, positive or negative, in the economic position of its Pakistani heritage community.

By the time the BiBBS cohort was recruited the proportion of Pakistani heritage women educated to A level or higher has gone up from around 37% in the BiB cohort members in the Better Start area to 49.5% in the BiBBS cohort. It may be that educational achievement imbues a sense of optimism about future economic security, replacing an historic assumption that one needs to rely on land or on the pooled resources of extended family. It may be that continuing in education pushes back the age at which women marry and it may be that educational achievement increases a sense of personal agency that transfers to partner choice. Continuing in education can be understood as both a reflection and generator of social change.

### Cultural factors

Consanguinity is seen as prioritising family ties that go beyond the couple who are marrying, marriage as a joining of families rather than of two people, the nuclear family norm that is found in many communities. Cousin marriage, it is argued, improves the chances of compatibility between the bride and groom’s families, makes marriage arrangements easier, provides an extended care and support network for children and for older people and provides a context to sustain and support all in difficult times. It also is consistent with a more general sense of wishing to maintain a particular linguistic and cultural identity.

In 2001 Shaw identified “arranged love marriages” which, while relatively infrequent, appeared to be increasing over time (arranged love marriages are likely to be convened between parents and may well be with cousins but the choice of partner has been influenced, or led, by the putative bride or groom. Arranged marriages and cousin marriages can of course also be, or become, “love marriages”.) It may be that we are seeing in the figures about falling numbers of consanguineous marriages and changes in the characteristics of those who do marry cousins a shift to “love marriages” presaged by Shaw nearly 20 years before but moving beyond the cousin marriage that was still dominant when her paper was published. Studying consanguinity may indicate a more prevalent affective individualism ( Stone, 1977) shaping a different sense of self and society, with a greater focus on one’s own wants and needs rather than a sense of a primary responsibility to follow a parental lead. Given our finding that the biggest fall in rates of cousin marriage is found in younger mothers (those under 25) it may be that we are seeing a generational change in this Pakistani heritage community.

### The importance of faith

There is a debate in Islamic scholarship about consanguinity and, for some, a sense that cousin marriage as a manifestation of faith may provide a motivation. Shaw (2001) and Al-Bar and Chamsi-Pasha (2015) summarise contrasting readings of religious and historical texts in Islam on this matter, underlining a contested and dynamic debate on the weight given to customary and faith based factors. In a Pakistan-based study Jabeen and Malik (2014) use the example of the Syed, who they term an “ethnicity/caste group”, and who they argue see marriage between close kin as a faith-based choice.

The sorts of relationships that are considered appropriate for marriage can be complex to identify. To help clarify what is forbidden in Islam there is a Quranic Arabic Corpus – an annotated linguistic resource for the Holy Quran. In this the 23 ^rd^ verse of chapter 14 sūrat l-nisāa (The Women) is considered and several interpretations by different scholars are offered. One, by Muhammad Sarwar, says: “You are forbidden to marry your mothers, daughters, sisters, paternal aunts, maternal aunts, nieces, your foster-mothers, your foster-sisters, your mothers-in-law, your step-daughters whom you have brought up and with whose mothers you have had carnal relations. It would not be a sin to marry her if you did not have carnal relations with her mother. You are forbidden to marry the wives of your own sons and to marry two sisters at the same time…. God is All-forgiving and All-merciful.” ( The Quranic Arabic Corpus - Translation : corpus.quran.com, accessed 30 Sept 2024).

As well as acknowledging the doctrinal complexities and the accretions of meaning over centuries of interpretation amidst changing social contexts and changing family structures we might also construe a debate within the Pakistani heritage community about consanguinity as being about social power. Perhaps young people’s changing Islamic consciousness and engagement with Islamic teaching has led to a questioning of practices that have long been promoted (by their parent’s and by preceding generations) but that they may see as equivocal in Islam. If these practices, including consanguinity, are reframed as cultural practices rather than faith requirements they are more likely to be replaced by a personal choice agenda.

### The relationship between Bradford and Pakistan

The examination of cousin marriage in Bradford, and everywhere there is a Pakistani heritage community in the UK, requires a consideration of the extent and nature of ongoing links with Pakistan ( Small, 2012). Cousin marriage often includes one partner from the UK marrying a cousin from Pakistan who would have moved before marriage or who would move to the UK to live around the time of the marriage. There are complex factors that shape the dynamics in this interaction. If economic wellbeing is an important factor then the relative situations extant in the UK and Pakistan at any one time may impact on the desirability of cousin marriage. If cultural and faith-based concerns are important then the congruence of attitudes between the two settings is important and if they are diverging that will impact on marriage choice (see Grjibovski *et al.*, 2009). There are also factors external to those shaped by the Pakistan heritage community, notably immigration laws.

Rates and patterns of cousin marriage in Pakistan, as reported in Pakistan’s Demographic and Health Surveys, have been relatively stable between 1990 and 2018 – varying between 63% in 1990–1, 68% in 2006–7 and 64% in 2018 ( Bashir & Nazir, 2022; Iqbal *et al.*, 2022). A study in Bhimber, a district of the administrative division of Mirpur Azad Kashmir, a part of Pakistan where many of Bradford’s Pakistani families originate, reported similar figures with 62% of total marriages being consanguineous and with marriage between first cousins predominant, these constituted 50% of total marital unions ( Jabeen & Malik, 2014.) BiB figures for 2007–2010 for Pakistani heritage mothers were 64% ( Wright *et al.*, 2013). BiBBS figures for 2016–2019 were 46%. We can see close similarities in rates in earlier data and then a more recent divergence. There is also a divergence in the characteristics of the consanguineous.

In reporting high and stable rates of consanguinity in Pakistan, Iqbal *et al.* (2022) identify consanguinity as more prevalent in young women, uneducated women, those living in rural areas, those with poorer wealth status and those with less exposure to mass media to access information. Pakistan’s *National Institute of Population Studies* (2013) reported that the probability of marriage outside the family increases with the woman’s level of education. In Jabeen and Malik’s study in Bhimber ( 2014) consanguinity was highest in younger populations which they conclude indicates “a time-dependent increase in consanguinity in this population” ( Jabeen & Malik, 2014 p309). It was also higher in rural areas. However, the picture diverges from Iqbal *et al.*’s country-wide data in that rates in Bhimber also increased with women who had more education, greater literacy and “greater economic status”. In any consideration of rates and patterns of consanguinity in Pakistan it is important to acknowledge regional variation and to be cognisant of the UK Pakistani heritage population’s historic links with specific parts of Pakistan.

In Bradford we report biggest falls in mothers under age 25, from 47% to 28%, while rates in the over 35’s were about the same in both Bradford cohorts. Rates also declined with longer time spent in education ( Small *et al.*, 2024a). Jabeen and Malik believe their study has *“implications not only for other endogamous populations of Pakistan but also for the sizable Kashmiri community immigrated to Europe”* ( Jabeen & Malik, 2014, p 301). But when we consider changing demographics of consanguinity we do not see a developing pattern in Pakistan that is likely to transfer to Bradford, rather what we see is divergence between the two (related) communities and a distinct aetiology for a Bradford population who appear to be changing in ways that are determined by a within country dynamic. Jabeen and Malik see an increase in consanguinity heralded by high rates in younger people, in Bradford we see the opposite – a decline in consanguinity most evident in the young.

Research in Pakistan has been concerned to consider the impact of changing fertility rates on consanguinity. The “availability” of unmarried cousin’s Mobarak *et al.* (2019) discussed (above) is linked to this. Shenk *et al.* (2024) also address the importance of considering fertility rates and the impact they have on the likelihood of their being cousins to marry, should such marriages be sought. But they also link the importance of considering fertility rates in Pakistan with the country’s broader economic position. “High rates of cousin marriage persist in Pakistan due to slow economic development which maintains motivations for cooperation with kin, and high fertility rates which sustain the large numbers of cousins that enable high levels of consanguinity” ( Shenk *et al.*, 2024, p1). Barakat and Basten (2014) ask what will happen to consanguinity if there is a “fertility transition” where the number of cousins eligible to marry declines? They suggest that consanguinity could diminish considerably, or features of it could change, it could become more coercive or it could be sustained by abandoning other social preferences, an older groom and younger bride for example.

### Importance of changes in Immigration Law

The 1971 Immigration Act was modified in 1997. This made it easier for UK residents to bring in a foreign spouse – non-European immigration rose substantially after this change. In 2012 new regulations were introduced to create a strict minimum income thresholds for anyone applying to bring a partner from outside the EU to the UK. This created greater challenges for inter-country migration for marriage. Our data highlights the importance of a reduction in consanguinity in those couples where both were born in the UK and a smaller but still significant decrease where one partner was born outside the UK but came here before the age of 16. Family Unification Visas issued for people from Pakistan to come to the UK have reduced – 10099 were issued in 2007, 7239 in 2012 and then, in 2013 the year after the Immigration Act, there were 3617. By 2019 figures had risen steadily and stood at 9288. ( Migration Observatory, 2023). Changes of this order may contribute to the reductions in consanguinity we report but in the period our data draws on are not of an order to constitute the major variable. It may be that if UK law continues to use increasing income thresholds as a means of control legal migration (in December 2023 a decision to increasing thresholds to £38700 was announced by the government) one likely consequence is that only those few in the Pakistani heritage group who are better off financially, and meet the required income threshold, would be able to have a spouse migrate to the UK. This is a policy that would exacerbate inequalities in opportunity in the Pakistan heritage community by discriminating against poorer families.

Consanguineous marriage is banned by law in some places. Bittles listed some states in the USA, China and Taiwan, North and South Korea and the Philippines ( Bittles, 2012: 31). More recently there has been a ban introduced in Norway in 2024 and a ban will come into effect in Sweden in 2026. A proposal to introduce a ban was made to the UK Parliament in 2024 but was not supported. A part of the rationale made by proponents of a ban in the UK was that this would be positive for children’s health. Opponents saw such a move as disproportionate (impacting on large numbers of people for doubtful health benefits) and ill-timed (when rates of consanguinity were reducing). Greater education about genetic risk and improved access to genetic testing, including couple-testing, were proposed as more effective responses ( *The Guardian* on-line 17th Jan. https://www.theguardian.com/law/2025/jan/17/tory-bill-to-ban-marriage-between-cousins-is-damaging-and-unenforceable?CMP=share_btn_url).

When we look at consanguinity globally we can identify other social circumstances constraining partner choice and thereby encouraging consanguinity. These include marriage choice in isolated rural or island communities, or in communities who feel themselves distinct from, or at odds with, the larger community they geographically live within (perhaps because of a strong religious beliefs or a distinct ethnic identity), or when social upheaval or war has divided a community from whom marriage partners have traditionally been drawn.

### Efforts to raise awareness of genetic risk

The Department of Health ( 2010 and 2012) and Khan *et al.* (2010) reported that, across the country, knowledge of genetic risk and service uptake among communities at higher risk of recessive conditions due to consanguinity was poor. By 2016 there was considerably more attention being paid to understandings of, and responses to, consanguinity and genetic risk (see Salway *et al.*, 2016 and Salway *et al.*, 2019). Shaw (2020) and Shaw and Raz (2015) have also explored debates on cousin marriage in the context of considering genetic risk and cultural change. ( Shaw, 2020; Shaw & Raz, 2015).

Health services in Bradford have long been aware of the health challenges caused by recessive genetic disorders and have sought to enhance health promotion and health education and to provide services to improve understanding of genetic risks as well as providing services to care for those with recessive disorders. Bradford Council and the NHS can refer closely related married couples to specialist genetic services for testing and counselling ( Bradford Child Death Overview Panel (CDOP) Annual Report 2017–18). The local NHS also supports raising genetic literacy in the population by providing information and advice directly or in collaboration with the voluntary and community sector. Expert input from *Genetics Communication Diversity* has been available across the city ( http:www.g-c-d.co.uk) (see Darr *et al.*, 2016 and Darr *et al.*, 2013)

BiB has been active in Bradford for a number of years. The size of the cohort and its longevity, together with its reliance on data from the local NHS and education services, ensure it is well known in these communities. It also has a high profile in the city via local media and via many outreach activities that keep cohort members informed about the study, it’s findings and its messages about health and well-being of the city’s children (see Quick *et al.*, 2017). These messages include those that followed the Sheridan *et al.* publication in 2013 about the link between consanguinity and recessive genetic disorder. This is a scenario in which a research study may enhance both professional and lay knowledge about health and so contribute to its improvement.

## Methods

### Why focus groups?

The relationship between information, understanding and action and the interface of behavioural and societal change are best understood by the sorts of exploratory discussion that are characteristic of focus groups. Understandings about what is changing, why these changes occur and what their significance is emerges discursively. A focus group can provide the space for exploration of complex and contested perceptions and the prompts used by its facilitators can ensure the subject of interest is kept central in the discussion ( Bloor *et al.*, 2001, p43). This research underwent an ethics review by the Research Ethics Panel at the University of Bradford and was approved on 31 August 2023. The reference identity number is E1112.

### How we decided who should be in the group – why the age and gender split?

We undertook focus groups with members of the Bradford Pakistani heritage population (the group that manifest the behaviour of interest and disproportionately exhibits the health and education problems associated with it.) We hypothesised that a generational and a gender-based difference in attitudes to cousin marriage may exist and also that group members might be more willing to discuss issues with those whose characteristics they most shared. Two focus groups were planned for men and two for women. The division by gender was made because of long experience of working in this community and experiencing the preference, most marked in older groups, to discuss in same gender groups. There would be two focus groups for young people who were in the age ranges between 18–26. One of these would be with young men and the other with young women. We considered these group members would be at an age where they would be contemplating marriage and would therefore provide insight into the factors they would be considering in reaching that decision. The second set of focus groups were attended by participants similar to the age of the young people’s parents’, a generation whose attitudes to cousin marriage are likely to have been formed when it was more commonplace than it is today and who may have considered they had an integral role in making marriage choices for their children. All of the attendees in the latter two groups were over the age of 50, one group of men and one of women. We note that the possibilities for cross-group discussion, including challenge, was sacrificed in making this choice about group membership. In total we conducted four focus groups with 7–8 people attending each group.

### Recruitment of participants and details of the groups

The recruitment was organised by SI who liaised with a local mosque, a Youth Club, the local University and a voluntary group. He invited people attending events in these settings to read an information sheet about the study and then, after some time for reflection and for questions, to complete a consent form (see material provided in our Extended Data, Small *et al.*, 2024). The only criteria for inclusion were that the person was from our target group, Bradford’s Pakistani heritage population, and that they consented to join the study. The decision to carry out four focus groups was pragmatic, shaped by research staff availability.

The range of sites for recruitment was likely to have made the study available to people with different interests and backgrounds. There is a high rate of attendance at mosques and the voluntary organisation we went to for recruitment is engaged in family support services including support for women. These are services that engage people from across the community. Youth clubs and University offer us a younger group and one that is attending these places for different reasons to those that lead to attendance at the mosque or support group. It is a range of sites that meant we could access people closely involved in the traditional structures of their community and those who were in settings where many different sorts of people were engaged alongside them. We recruited people from a variety of Pakistani cultural backgrounds (e.g. Pathan, Pakistani and Mirpuri backgrounds) and people spread across the city rather than concentrated in one area of Bradford. Some of the participants knew each other but most did not.

The focus groups were conducted by SI and RR. Both are of Pakistani heritage and have experience in conducting qualitative research. SI & RR facilitated the focus group with men over 50. Both of the younger adults focus groups were facilitated by SI and the group with women over 50 was facilitated by RR.

Each focus group lasted 90 minutes and commenced after the facilitators had checked participants had read through the participant information sheet and then had completed and signed an informed written consent form (forms are available in this paper’s Extended Data (see Small *et al.*, 2024). All focus groups were audio recorded and were transcribed verbatim. The Participant Information sheet indicated that access to transcripts would be restricted to the research team and material which might identify individual participants would not be shared beyond this team.

### Topics for the groups

We started our focus groups by describing what cousin marriage is and what the recent data is telling us (in terms of decline in rates and the identification of more frequent adverse health and education outcomes for children of consanguineous parents). We asked people to reflect on their own observations and to discuss whether or not the data resonated with their experiences. The focus group then followed up with a series of questions which the research team had formulated focussing on the cultural, political and legal systems which may explain changes in the rates of cousin marriage and on ways adverse risks could be best communicated (see Small *et al.*, 2024). The questions, staying within the spirit of focus groups, were asked in a way that stimulated interaction between participants. All the focus groups followed the same schedule and researchers ensured that all participants were able to contribute to the discussions. Everyone appeared willing and candid when sharing their opinions and it required little prompting from the facilitators to generate dialogue. None of the questions appeared difficult to answer. We were reassured as to the salience of the topics chosen and encouraged by the willingness of group members to engage.

### Details about the focus groups

The focus groups were conducted in English. It is possible that people either declined to be involved in the groups or contributed less than they might have wished to because of a lack of confidence in speaking in English in a group setting. We mitigated this risk by having focus group facilitators fluent in the languages most commonly used in the Bradford Pakistani community and offering participants the chance to speak in the language they felt most comfortable with. It is possible that even if people agreed to join groups being undertaken in English that they felt inhibited in contributing because they thought their English was not good enough. Our experienced facilitators would have been alert to this, but we cannot be sure that nobody was put off contributing.

Focus groups were held in a variety of venues including the local hospital’s research centre, the University and a Youth Centre.

- For the younger men’s group, most were either on a training scheme/apprenticeship or were in-between completing college and either starting University or seeking employment.
- For the younger women’s group, most were completing their final undergraduate year at University, with one participant who was employed as a freelancer, having completed University.
- For the over 50s men’s group, most were either self-employed or held a job with an employer. Two men had recently retired.
- For the over 50s women’s group, most participants were either homemakers, carers or volunteers. Very few were in paid employment.

The focus groups are identified in our findings as, older men (OM), older women (OW), younger men (YM), and younger women (YW). We also include line numbers from our transcripts.

### How focus groups were analysed and how themes were developed

A constructionist, critical, and inductive framework guided this analysis ( Byrne, 2022). The constructionist approach was well-aligned with the study’s objectives. It facilitated an examination of how societal, cultural and contextual influences shaped participants’ understandings, attitudes and behaviours. Our critical orientation, sought to identify and analyse broader societal structures that were reflected in the participants’ narratives.

The data corpus for the analysis were the transcripts from the four focus groups which were analysed using Thematic Analysis, based on Braun and Clarke’s 6-step framework ( 2006: see also Braun & Clarke, 2022). This framework is an *analytic process*, not a rigid method, it has creativity and reflexivity at its core. Revisions and changes are possible throughout. Braun and Clarke make it clear that themes do not emerge from the data, instead the researcher actively constructs each of the themes through their engagement with it, and through their discussing any divergence of opinion ( Morriss, 2024). The approach is both inductive (bottom up) and realist.

Analysis was undertaken and data were coded (separately) by the authors AC, VS and ZK. Each researcher began by independently reading the focus group transcripts multiple times to familiarise themselves with the data, making detailed notes and observations. The coding stage commenced with each researcher coding the transcripts line by line. Subsequently, the analysts convened to discuss their initial codes, ensuring shared understanding and consistency. A traditional pen-and-paper approach was used to document and categorise codes, with post-it notes serving as a physical medium for organising and refining ideas.

Once the analysts agreed that all contributions had been coded, the codes were reviewed to identify themes and sub-themes. Themes were constructed using Buetow’s (2010) saliency criteria, which assesses the frequency (the recurrence) and the importance of themes. Buetow defined importance as themes that “advance understanding or are useful in addressing” the study questions. Frequency alone does not indicate importance; an important theme may not recur often. It is the analysts’ interpretation which determines which codes are and are not important ( Braun & Clarke, 2006).This approach allowed the researchers to prioritise the most significant discussions for inclusion in the results while also recognising divergent or less frequently expressed perspectives. These could be presented as sub-themes. The focus was on capturing both the dominant narratives and nuanced insights so ensuring a comprehensive representation of the data.

Analysts worked independently to identify patterns, merging and collapsing similar codes into potential themes. This iterative process was repeated across all transcripts, each of which represented one of the four focus groups. Following this, analysts reviewed and refined the themes to remove those lacking sufficient support and to consolidate overlapping content areas. Where there was disagreement regarding the coding, the authors engaged in discussion and adjusted the coding, where relevant.

Three main themes and nine sub-themes were identified through this process. Each analyst was responsible for synthesising one theme into a detailed write-up. Themes were cross-checked against each other and the original dataset to ensure consistency, distinctiveness, and alignment with the data. Relevant quotes from participants across all four focus groups were incorporated to provide rich, illustrative examples. To ensure transparency and enhance rigour, the analysts participated in regular peer debriefing sessions. These meetings allowed for critical reflection on the evolving analysis, the questioning of assumptions, and the comparison of interpretations with the original data. Triangulation was achieved by having all three analysts independently code the data, while their lack of involvement in data collection ensured an unbiased approach, free from preconceived assumptions. The team’s diverse backgrounds, including members of the Pakistani heritage community and others with experience of the study setting, brought a range of perspectives to the analysis.

The analysts reviewed each other’s write-ups to ensure coherence, and one analyst integrated all themes into a results section, paying close attention to consistency, flow, and clarity. The other authors (NS, RR and SI) then considered and modified this analysis and all authors reviewed and approved the findings presented below. ( Table 1: Summary of themes and sub-themes).

**Table 1.** Summary of themes and sub themes.

Theme	Sub-theme	Theme components
**1.** The influence of religion and of changing social contexts on marriage choices	**1.1:** The importance of faith.	• Faith as a guiding principle in marriage decisions and family unity • Varying interpretations of religious teachings • Balancing religious values with changing societal and personal preferences
	**1.2:** ‘Moving with the times’.	• Increased acceptance of love marriages and autonomy in partner choice • Younger generations diverging from traditional practices • Tensions between maintaining traditions and adapting to societal change.
	**1.3:** The right age to marry.	• Prioritising education and career before marriage • Delaying marriage to achieve financial stability and independence • Tensions between family preferences for earlier marriages and individual readiness for marriage
	**1.4:** Economic, political and legal changes - impacts on consanguinity	• Improved economic independence encourages broader partner selection • Changes in immigration laws • Increased awareness of risks associated with consanguinity
**2.** Finding safety in tradition	**2.1:** When marriages go wrong.	• Breakdowns in consanguineous marriages can create significant challenges
	**2.2:** More ways to meet a potential partner	• Diversified ways to find partners due to growth of digital platforms, social media. • Young people increasingly prefer self-initiated relationships.
	**2.3:** Consanguineous marriage as a “fallback” plan	• Cousin marriages seen as a reliable option if other routes fail • Perceived as a safer choice due to family trust and cultural compatibility
**3:** Risks associated with consanguinity and ways to make people aware of them	**3.1:** Awareness of the risks associated with consanguineous marriages	• Growing awareness of genetic risks through health campaigns • Personal experiences with genetic disorders in families • Increased engagement with healthcare professionals for advice and screening
	**3.2:** Strategies to encourage discussion and to educate about consanguineous marriage and genetic risk.	• Need for culturally sensitive health education initiatives • Use of religious leaders and respected figures to foster conversations • Promotion of genetic counselling as a non-stigmatising, supportive resource

## Findings

### Theme 1: The influence of religion and of changing social contexts on marriage choices

Across the groups, there was acceptance that consanguineous marriage was undergoing changes in this community. Theme 1 captures the factors that group members link with changing attitudes and social practices. There was significant difference between the groups, age and, to a lesser extent, gender underlay these differences. Overall, the differences are characterised by a concern with what a shift from consanguinity would mean for their community. We present data according to sub-themes looking at reasons for and against consanguineous marriage, changing social practices, and the impact of the political and legal context. (See Table 2 for a Glossary of terms used in the focus groups.)

**Table 2.** Glossary.

Word used	Meaning
*Allah Subhan’a Tallah*	May He be praised and exalted
*Al-Nisa*	An-Nisa' (meaning: The Women) is the fourth chapter (sūrah) of the Quran, with 176 verses (āyāhs).
*Āyāh*	This means verse from the Quran
*Biraderi*	Kinship and brotherhood
*Chacha/chachi*	Uncle / Aunt (more precisely - Chacha is paternal uncle and Chachi is his wife).
*Fitnah*	Perplexion, deceit and misguidance.
*Hindko*	A language spoken by people of Pakistan and Afghanistan. It is commonly spoken by people in Northwestern Pakistan
*Mehram*	Refers to a person with whom marriage is prohibited because of their close blood relationship, because of radaa'ah (breastfeeding), or because of being related by marriage.
*Muqaddar*	Destiny/fate
*Pathan*	An ethnic group spread all over South Asia who have distinctive culture and customs.
*Tarakhelli*	One of the Pathan tribes

***Sub-theme 1.1: The importance of faith***

The group made up of older men foresaw a fall in rates of consanguinity as something that would dilute their culture and tradition; the importance of religion was emphasised:

*“Allah Subhan’a Tallah knows what’s good for us and what’s not good for us and therefore He gives us these guidelines on what is allowed in terms of marriage – who we can marry and who we can’t marry.” (OM, 484–486)*

*“I printed off an ayah and there’s a sole chapter isn’t there, a section/verse from the Al-Nisa, in chapter 23…. So, Allah Subhan’a Tallah has, given us guidelines.” (OM, 487–49)*

The older women had similar view about the importance of religion:

“ *We believe that you know what our Prophets say, and we follow that. So, if it wasn't right within ourselves, we would have been told not to*” (OW, 236–238).

A resort to destiny, or fate, can allow you to reconcile a faith based position with different choices:

“ *It's allowed. And about destiny, you know, like, your fate is in your hands. You can't say it’s Allah's will. Allah has given you that choice to make it possible*” (OW, 60–608),

The impact of religion was mentioned by the members of the two focus groups of younger people only in so far as their asserting that they would marry a person of the same faith.

***Sub-theme 1.2: ‘Moving with the Times’***

There was a sense in all groups of shifting understandings of how family is defined and how power dynamics within it are changing.

The older women’s group perceived their children were considering marriage choice using a different set of understandings about what constitutes a close family relationship, they had a different view of the resonance of being a cousin.

*“My children who have got a different perspective, they would call them [cousins] as brothers and sisters.” (OW, 11–13)*.

This group member agrees, but she is arguing that this cultural change does not change the religious edict on this matter:

“ *so, they class it as, like, say that she's my sister, he's my brother. Islamically, really they are your mehram’s they’re non-mehram because, you know, you can marry them. But they don't see like that” (OW, 18–20).*

The cultural change is vividly captured in the next two quotes. First, a younger women talking about the idea of marrying a cousin said, *“ew, that’s like my brother!” (YW, 33)*.

Then, in this focus group we get an insight into peer pressure to move away from consanguineous marriages:

*“I feel like amongst your friends, it's more strange to say that you're marrying your cousin” (YW, 30).*

Nevertheless, the balance of opinion in the focus groups was that parents should have an important role in who their child marries.

*“Parents, they do have a role. Like, to see who you’re marrying, to see who the family is, you know? If they’re suitable, if they're a decent family” (YM, 135–142)*.

Younger women felt that if they married outside the family they risked being seen as leaving it:

*“Because in our family it’s kind of like the girl gets married outside, then it’s kind of like, ‘she’s gone away now that she’s not going to be part of the family’” (YW, 137–139).*

Both younger groups believed that they would disappoint their parents if they married without their approval:

*“You don’t want to disappoint your parents” (YM, 595)* and [there is a benefit of] *“having the guy contact your dad, and your parents giving like the permission for you guys to get to know each other, like openly with them involved in the conversation” (YW, 111–112).*

There are some things that make that approval more likely:

*“My mum, she's always said that as long as they're a good Muslim... My family the priority always is that it has to be a Tarakhelli like me, like in our tribal system (YW, 134–137).”*

A more sceptical view of the parental role was made in the younger men’s group:

*“but they’ve still got that say, you know, ‘that family’s not good’. They'll make politics up.” (YM, 164–165).*

Perhaps consanguineous marriage is a way for the older generation to exert control over the younger:

“ *often it's a case of pushing your daughter or your son to a form of control as well… we sort of think he's off the rails a bit, let’s get him married to somebody from Pakistan, she might straighten him out” (OW, 43–45)*.

All groups described changing social trends impacting on choice of a partner and reflected on the decline in consanguinity. However, there was a noticeable divide between the older and younger generation. This concern with the impact of “moving with the times”, was observed amongst the older women:

*“What does ‘move with the times’ mean? Because that's sort of conflicting with my protected characteristic, which is faith …when we think it’s culture it’s family's, it’s tradition, you have to marry within the family, but that thought is changing.” (OW, 403–409)*

A part of the “changing times” groups discussed related to links with Pakistan:

*“years ago, people would get married majorities from Pakistan, would be the reason for that marriage, would be to bring somebody over to this country” (OM, 557–559)*.

Our younger women objected to this sort of strategy:

*“I feel like say like, we go to Pakistan as like young British girls, and they'll just see you as that passport.” “Yeah, they don't see the personality, they just see the base and the passport” (YW, 335–339).*

Another part of “changing times” was the impact that expanded opportunities in higher education were having on the relationship between the generations.

*“they are going through universities in higher education, they're suddenly finding themselves, they're middle class, they don't want anything to do with the poor people in the family” (OM, 170–173)*.

***Sub-theme 1.3: The right age to marry***

The two younger groups described wanting to find their own right time to marry:

*“I know a couple of girls who are not married, and they were just like, ‘no, we wanted to focus on education’, and then marriage will come afterwards” (YW, 582–583)*

*“it’s your choice at the end of the day as well, whenever they feel like it's right time and whenever they feel comfortable” (YM, 33–35).*

There was, however, societal pressure about when to get married, the younger women felt they:

*“needed to be married by a certain age or they were sort of like a burden on their parents.” (YW, 259– 260)*

*“For a girl in Pakistani culture if she's past 25, then it's a bit of a stigma that ‘why is she still studying, why isn’t she getting married?’”. (YW, 171–175)*

*“I feel like guys generally, especially in the Pakistani culture, they have a lot more freedom and autonomy to make their own decisions”. (YW, 267–268)*

In theme 2 (below) we discuss how a consanguineous marriage might be seen as a “fallback plan”. If, by a certain age, a woman has not married the family can step in. Here the older men’s group were critical of the expectations of those looking to pick their own marriage partner:

*“You come across a lot of people they’re not married, especially women, they're finding it difficult to find partners, you know, because of their attitude towards the calibre of people that they interact with. They’re trying to find that right person”. (OM, 273–276)*

The older women commented on a tension between exercising individual choice and a cultural pressure to obey one’s parents:

*“I was forced into a marriage. And we couldn’t call it forced marriage…. it was like disrespecting our parents.” (OW, 469–470)*

***Sub-theme 1.4: Economic, political and legal changes - impacts on consanguinity***

Although all groups addressed potential political and legal reasons for the decline in consanguineous marriages, it was predominantly the male groups who perceive these as significant issues. The older men described the financial implication of bringing family members to the UK, suggesting that new rules and higher costs were preventing consanguineous marriages:

*“We’re still looking into it, my sister-in-law …it takes about 3 to 6 months. She was saying that the charging her £1500 for the passport” (OM, 508–511)*

The younger men emphasised policy changes, Immigration Laws in particular, as being the factor limiting marrying people from Pakistan:

“ *it’ll close the opportunity of bringing someone over or going there*” *(YM, 201–205)*.

These younger men did not appear to be too concerned with the impact of this:

“ *from my point of view, it’s going to affect the back-minded people, they're just going to be panicking that they can’t get our relatives over, but I think now the new generation, it will be okay for them because they'd be like, we've not got thirty eight grand [£38000] to bring them over*” *(YM, 208–210).*

Discussion around the place of land ownership in marriage choices saw differences between older and younger groups. Older men were fearful that a shift from tradition risked losing the land that has been in their family for generations. The older women did not mention land, or financial need, as a reason to get married. Younger women, however, expressed strong opinions:

*“I feel like especially because we're like British born, we don't really care about land back home” (YW, 277–278).*

“ *We’re all kind of sick of it, like you don’t see that land. No one is using it.”, YM, 291–292)*.

### Theme 2: Finding safety in tradition

Participants in all four focus groups discussed the potential benefits of consanguineous marriages and why the tradition existed. However, there were differences in perceptions between the groups. The older men focused on the benefits of having support from the biraderi as well as support from extended families. In arranged marriages (including consanguineous ones) both partners’ families already know each other, and it is assumed that the couple can rely on family support to resolve issues. They saw “love marriages” as manifesting the couple’s need for independence and autonomy which was seen as a sign of them drifting away from the safety and support offered by tradition:

*“Yeah, because then you’ve got your arranged marriages, you’ve got your family support, you’ve got your biraderi support, and everybody knows each other and their backgrounds”. (OM- 418–420).*

Older men and older women felt that it was their duty to protect their families, especially their daughters. They saw consanguineous marriages as offering a robust vetting process for the potential spouse and their family, thereby reducing the risk associated with marrying into an unknown family:

*“She was saying that (…) ‘I trust my sister’s son to look after her daughter’” (OW, 95–96)*

*“If I had a daughter, I’d certainly get involved in her marriage choices, just to protect her modesty, basically, because I’m fully aware of the sort of fitnah that are out there. And I don’t want my daughter going on the internet searching for a spouse, and then the sort of things that men can do”. (OW, 492–495)*

Older participants also spoke of people who married outside their family regretting their decision:

*“I've got friends who are like in their 40s now, and they're saying, ‘you know what, we're isolated. We wish we did marry into the family’.” (OW, 199–201)*

A concern about possible social isolation featured in the older women’s group, with some associating this with a decline in consanguineous marriages:

*“I think, because the more cousin marriages are declining, the more social isolation is increasing.” (OW, 28–29)*

The younger men and women understood the perspective of the older generation that consanguineous marriages can offer more safety and security through familiarity with culture, values, language, and traditions. They also appreciated an argument that it led to increased social cohesion and integration within the family. But members of the younger women’s group saw ways these positives could be achieved without cousin marriage:

*“For the togetherness it’s good (consanguineous marriages), like keeping as a community within it” (YM, 273)*

Others talked about how they would support their spouse to integrate with their family if they were to marry outside their family or language group:

*“I feel like I still want like a Pathan Muslim guy just so we speak the same language and everything”. (YW, 145)*

“ *I think I’d have to give him some Hindko lessons” (YW, 150–151).*

***Sub-theme 2.1: When marriages go wrong***

The older men focused on the positive aspects of consanguineous marriages; negative consequences did not present as major concerns. The other three groups considered positive and negative implications of consanguinity. The negative implications were identified as important explanations for the fall in consanguinity rates, particularly by the younger men.

In a consanguineous marriage, the impact of a failed relationship in one couple can reverberate throughout the rest of the wider family, leading to a deterioration of family relationships:

*“When the problems exist, it breaks both sides of the family in big ways.” (OM, 431–432).*

*“Because I think like growing up we’ve seen marriages, and it just ruins the family, ruins a family dynamic. And not only as a marriage gone there, but you've lost your relationship with your cousins, other families” (YM, 220–221).*

*“I think that's why people don't want to marry in the family anymore. Because it just ruins the rest of the family” (YW, 213–215).*

***Sub-theme 2.2: More ways to meet a potential partner***

All focus groups discussed how, in the recent past, there was limited interaction in the community between men and women. Group members discussed how first-generation immigrants were more deeply connected with their families in their home countries and less integrated within their new UK context. They preferred to get married in their home country, and such marriages would usually be consanguineous. However, over the years, this has changed. The younger generation’s social networks are wider and, in addition, social media such as Snapchat and Instagram as well as dating apps and websites have made it easier for young people to interact without the involvement of families:

*“I feel like young Muslims are just starting to like, connect more with each other guys and girls, it's just more easily accessible for you to find someone say at university, or just like randomly, through friends rather than like, going out and try and seeking your cousin for your family” (YW, 46–49).*

*“Find each other social media, you know, all these kinds of messages. Quick adds. Quick adds on Snapchat” (YM, 109–112).*

Despite this, there are still examples of younger people who choose to marry their cousins. Participants in all focus groups highlighted the importance of honouring someone’s choice of a partner and not vilifying consanguineous marriages.

*“And the last thing you want is to marry someone you've grown up with. But they decided because of, like you were saying, safety”. (OM, 91–93)*

***Sub-theme 2.3: Consanguineous marriage as a fallback plan***

Younger participants, both men and women, perceived consanguineous marriages to be a safe backup plan when efforts to find a partner on your own had failed.

*“ ‘I’m going to go back home and marry my cousin’, it’s just a thing. It’s Plan B.” (YM, 560–561).*

Older women referred to their cultural role as parents including finding a spouse for their child. Marrying a cousin would be better than not marrying at all, once other options have been exhausted:

*“It becomes a concern for a parent. If you can't find someone, then what choice do you have? You have to knock on the door of your cousin?” (OW, 674–676).*

Older men however were proponents of consanguineous marriages being the first option. There were community and safety benefits. They believed that young people overlook this safe option only to regret it later:

*“I find our family a lot of them have been like, you know, you're going to marry your cousin. And then ….they're going into education and getting older. …I'm not going to get married there. I think that happens a lot now. And then the struggle… to actually find a partner” (OM, 290–293).*

### Theme 3: Risks associated with consanguinity and ways to make people aware of them

Across the focus groups, there was discussion regarding the potential risks associated with consanguineous marriages, and about how common risks were. Alternative, or additional, explanations for genetic anomalies were offered. There was discussion about what people could do to minimise risk, being screened for example. There was some challenge about the validity and significance of statistics, particularly within the older men’s group. This drew the conversation towards the topic of how risks should be discussed, and who should lead this discussion. Proposed strategies differed between the generations and genders.

***Sub-theme 3.1: Awareness of the risks associated with consanguineous marriages***

Across the four groups, there was awareness of the potential risks for children from consanguineous marriages. This was largely based on anecdotal evidence from group members own experience or the experience of others they knew. This was particularly evident as a subject of interest in the older women’s focus group:

*“The biggest thing in our family was when one of my cousins married his second cousin, and their child ended with a mitochondrial disease, and we'd never heard of that in our family. It was something that was, you know, just out of the ordinary.” (OW, 264–267).*

*“And then my dad's elder brother and his wife, again, were first cousins. They've got children who've got problems with speech, their voice, throat thing. Then my in-laws, which is my dad's brother again, they've got two sons - one's got epilepsy, and they've got some condition, I don't know what it would be diagnosed as here, we don't know what they call it. But my dad’s sisters are all married outside of the family and their children are perfectly fine.” (OW, 151–155).*

*“My mum and dad are first cousins - I’ve got two sisters that have got cerebral palsy, my brother had a hole in his heart when he was born. My chacha and chachi were first cousins, their children have got dwarfism.” (OW, 145–147).*

There was a hesitancy about attributing all problems to consanguineous marriages. The older groups wondered about other causal factors, perhaps being cousins was more of a coincidence than a cause:

*“I've got three children with special needs - two have got autism, and one's got epilepsy, and learning difficulties. They’ve had the genetic tests, and they’ve come back negative, the doctor can’t see what the reason is.” (OW, 178–180).*

This older men’s group pushed back against attributing adverse health outcomes to consanguinity:

*“The various disease that comes from genetic, you know, what they’re researching now, it happens everywhere not just in Pakistanis. It happens with Indians it happens with English people as well.” (OM, 670–673).*

*“In my family, I think about at least about 6/7 people in my family are married to cousins, yeah, even I was married to my cousin first. My brother was married to a cousin, we’ve never had deformities, or any other diseases or anything due to this genetic research.” (OM, 698–701).*

*“What about back home in Pakistan? If you go back and you know we all come from villages, yeah, every other person is married to first cousin, and there isn’t a lot of deformities or anything as such. You know what I mean and they’re all healthy, no medical conditions, nothing whatsoever.” (OM, 725–728).*

This view was supported by the older women who, like the older men, referred to the fact that consanguineous marriages have been occurring for many generations:

*“You’ve got to remember, these people came from families that for generations, they were doing the exact thing, and you know, what has caused it? It can't just be one factor. There has to be multiple reasons behind that. And that's what we need to encourage researchers to look into really.” (OW, 256–259).*

When the older men’s group engaged with the risk statistics they remained sceptical:

*“Is 6% something to be worried about? I don’t think that's a very high percentage really...” (OM, 825–826).*

*“This gene thing is only 6% of it, 94% is all these other things, like I said finding the muqaddar.” (OM, 949–950).*

The younger groups showed an awareness of risk and an acceptance of its association with consanguinity. Discussion focussed more on the steps one could take to minimise risk, such as pre-martial testing. Additionally, amongst the older women, there was mention of how the results of testing had influenced their decision about having children:

*“I know of someone that married the first cousin and before the engagement or anything even happened, they both got health checks done, just to be aware that are they carrying like any genetic diseases that might be passed on to their kids. And I think that's really important, like whether you’re marrying your cousin or not to have an open conversation about that within the Pakistani community. Just as like a pre-question before you get married to someone.” (YM, 420–425).*

*“But I know with my cousin, what they decided when they found out that, they have one son who was normal, had no issues, but their second child had the mitochondrial disease condition. And so, with them, they then decided to do the genetic tests, and they said one in four children would end up being of similar kind of health. So, what they decided, they wouldn't have any more children.” (OW, 430–436).*

***Sub-theme 3.2: Strategies to encourage discussion and to educate about consanguineous marriage and genetic risk***

When asked about how current and future generations could be educated about the risks of consanguineous marriages, there was a clear distinction between the generations, and between genders in the younger groups. The older men were concerned about what information is shared, how it is shared, and by whom. There was a general dislike of schools informing children about risks. Instead, they preferred information to be passed on by medical professionals, who, in their minds, would present the data in a balanced manner. Again, amongst the older men, there was a perceived need for control over how and what information was shared, with a concern that if given incorrectly, the information would *“demonise”* the traditional approach of consanguineous marriages, and *“brainwash”* the children with misleading information:

*“Like for example this study came in and then quietly the teacher from the classes in primary and secondary school, they started telling kids the deformities and disabilities were happening because of this.” (OM, 81–83).*

*“What’s important is this, how the data is acquired, how it's presented, who presents it? What's most important is that a particular community is not demonized with it. A particular community is not pushed and is basically that this community is doing this, because of this. And people should be given the opportunity of having access to the information.” (OM, 955–959).*

The older women’s group suggested the parents should have that conversation about risk:

*“What you do is that you have these discussions with your children of a young age” (OW, 189–192).*

The younger men’s group had a range of ideas about who should talk about risk. There was a role for the elders in a community, for a trusted source, and “ *someone from a south Asian background*” (YM, 493), with the information being shared in a trusted location:

*“I think we can do it in like community centres, mosques.” (YM, 496).*

*“People are more likely to take the information if it's coming from somewhere they trust. We can do that within our community. There's plenty of people who are.” (YM, 499–500).*

The younger men had the most varied suggestions about targeting different groups and about different means of communication. These included government websites, schools, and health professionals, as well as social media, such as Facebook, Instagram, TikTok, and Snapchat, and targeting certain methods aimed at specific group, e.g., “ *WhatsApp Aunties” (YM 439*) to engage with older women who share information via text messages.

*“Or maybe even if you go to your GP, you know, one of your local doctor's surgeries? And yeah, if it's advertised over the leaflet or poster or maybe even you know, on .. billboard.” (YM, 458-459).*

## Discussion

This section brings together the research that prompted this study, the literature reviewed in the “Why cousin marriage?” section and the findings of our focus groups.

There was agreement across our focus groups that rates of cousin marriage have reduced. But there were differences about the significance of that reduction and the reasons for it happening. There was not such a consensus about links between consanguinity and genetic illness and links with wider morbidity were little known. There was concern about how genetic risk was discussed and about the dangers that an enhanced risk could be used to criticize a whole community rather than inform about a particular social practice.

### Fall in numbers of cousin marriages

There was considerable discussion about social change, about cousin marriage in an Islamic context and about the changing relationship between Bradford and Pakistan. Shifts in patterns of education and changing immigration laws were also considered in our groups. There was a notable age difference with the older groups much more concerned about the impact of changing marital choices on tradition and community cohesion. There were some differences according to the gender make up of both the older and younger groups. In the younger women’s group there was a particular focus on the expectations women felt they were subject to.

The link between faith and cousin marriage was interpreted differently in the older people’s focus groups. Older men spoke in terms of a close link between cousin marriage, culture and tradition. They felt a move away from cousin marriage was a threat, not least because they spoke of this sort of marriage as being supported by the dictates of faith. Much as we have discussed in our review of literature there were different understandings as to what was required or allowed, participants spoke of guidelines, or in some cases prohibitions. Sometimes positions discussed in the same focus group were contradictory, in the older women’s group for example, one member said, *“Cousin marriages are forbidden”* and another said that cousin marriages are *“allowed”*. In that same group one person spoke of how Allah had given choice to ensure *“your fate is in your hands”.* The link between faith and cousin marriage was not discussed in the younger groups, save for their members expressing a clear commitment to marrying a person of the same faith.

The discussions on faith sat closely with a sense that a decline in cousin marriage was part of a more general cultural change, a *“moving with the times”*. The member of the older women’s focus group who expressed a concern with what *“moving with the times”* meant for what she called her *“protected characteristics”*, her faith, culture and tradition, perhaps best summed up the concerns that seemed widespread across the older groups. There were differences between the generations about how, why and when one chooses a marriage partner. Contrasting views on the merits of marrying a cousin were accompanied by some differences about the age at which one should marry and how quickly one should have children. These are differences played out in many cultures but here they occur in the context of cousin marriage being seen by our older focus group members as a litmus test for a shift from tradition. Such a shift makes our older focus group members anxious. The younger people’s groups were characterised by caution, they wanted a change but did not want to lose a sense of family and community support for their choice of partner and for their subsequent married lives. Younger people valued some aspects of change, choosing ones’ partner, being able to continue in education, or not being expected to marry someone from another country who they did not know, but they also wanted parental support for their marriage choices, they saw the value of extended families and recognised the extra pressure on young couples who did not have this support. Older people saw their children going into higher education and becoming different, finding themselves as *“middle class”* people who *“don’t want anything to do with the poor people in the family”*. But our younger people did not appear to want to separate, it’s more of a gentle rebellion if it is characterised as *“focus on our education and then marriage will come afterwards”* or that, *“parents, they do have a role”*. Older groups saw cousin marriage as a preferable choice because it emphasises a commitment to tradition and it has benefits in terms of social support, younger groups felt that tradition and support were important and could be achieved without cousin marriage.

The members of our younger groups saw cousins as more akin to siblings and, as such, not to be considered as marriage partners, even though their parents felt cousins remained acceptable, in some cases preferable, marriage partners. Designating who was *“mehram”* or *“non-mehram”* differed according to age-group. Peer group norms in the younger group have shifted. One of the members of our younger women’s group said it would be *“strange”* to tell your friends that you are marrying a cousin. This redefining of the family by younger people is not a conventional shift from extended family to nuclear family that is associated with modernisation in the sociological literature, rather it is a reclassification of cousins into what these younger people are defining as the closer family. It is a de facto setting aside of cousin marriage via a new lexical semantics – an assertion of a relationship as taboo in a way that might avoid too much head-on conflict with their parents.

There was discussion about cousin marriage as *“a fallback”* if a partner was not found. In the older men’s focus group they spoke of the difficulty some experienced in finding a partner – perhaps because the person (the woman) was too choosy, *“They are trying to find the right person”.* Assistance by the extended family in arranging a marriage can overcome that difficulty. The idea that prioritising trying to find the right partner can be problematic is a position at odds with a wider cultural assumption of the centrality of romantic love in marriage choice. Romantic love can be reframed in an arranged cousin marriage as marriage first, love later.

As we surmised above any influence economic wellbeing had on changing marital choices was hardly discussed. When it was it underlined a marked difference between generations. For example, in the younger women’s group the perceived importance given to land ownership in Pakistan by their parents was dismissed, *“we don’t care about land back home””, “We’re all kind of sick of it…No one is using it”.*

If these family dynamics are being enacted in a way that is seeking accommodation between the generations a shift in the nature of the relationship between the family and community in Bradford and in Pakistan is more sharply drawn. The older men’s group identified the importance of a motivation for marriage in the past as being to *“bring someone over to this country”*. Younger women objected to this sort of strategy, *“we go to Pakistan …and they’ll just see you as that passport”*. In our literature review we discussed a divergence between Bradford and Pakistan in terms of patterns of marriage choices and also considered practical barriers – changes in immigration laws for example. Our groups reflected on the cost of arranging migration, visa costs, as well as on the impact of much higher income thresholds that must be met if someone is to move to the UK. Our younger men’s focus group thought the cost pressures would limit opportunities to bring someone over, but participants did not see this as a problem. Here we see another clear distinction between generations, summed up by our younger men’s group participant who said these cost pressures are *“going to affect the back-minded people”* who will be panicking if they cannot bring relatives over. This younger man even intimated at some relief that the *“bringing someone over”* option might have disappeared, for *“the new generation it will be ok for them because they would be like, ‘we’ve not got thirty eight grand to bring them over’ ”.*

In addition to discussion on the resilience of tradition and of the impacts of social change, the expansion of involvement in education and the assertion of individual agency over choice of partner, we also see another influential area of social change, the impact of technology. In the past marriages might have been arranged by extended families and by biraderi networks or even by *“marriage brokers”* who have been involved in the community for generations. But now our younger people’s group members tell us they have the opportunity to *“find each other on social media…quick adds on snapchat”* as well as expanded networks for those in higher education meeting peers, or through friends, rather than *“seeking your cousin like for your family and stuff”*.

This is a hybrid assimilation into a wider social norm, hybrid because it involves individual choice but a choice that assumes the benefits of “within group” solidarities. The partner the young person chooses will be a *“good Muslim”* from a *“good family”*, in some cases, they will be from within their “ *tribe*” and, as such, this would be a partner who will not disappoint your parents.

### Genetic risk and public understanding

Much of the discussion about consanguinity and excess mortality and morbidity in childhood has conflated a cultural choice – marriage with blood relations – and a genetic risk. This risks confusion caused by an association eliding into a cause in non-specialist discourse. This confusion can have an impact on health promotion, health education and the take-up of available services when people from the community who customarily have blood related unions feel that not only this choice but this choice as a signifier for the general cultural constellation they occupy is being criticised. If you can separate cultural practice and genetic risk and focus discussion on the latter then insights are more easily understood, accepted and acted upon (see Darr *et al.*, 2013; Darr *et al.*, 2016).

Those parts of our focus groups that consider genetic risk and how best to discuss it well illustrate the challenges. The presence of anomalies and abnormalities in the Pakistani heritage community was recognised by all, many participants had stories they shared from their own family or from those they know well in the community. But this recognition was tempered by knowing that many families did not have children with abnormalities even though the parents were cousins (in the UK and in Pakistan). This prompted a belief that cousin marriage could not be the only cause of abnormalities and illness in children, *“there has to be multiple reasons*”. There were different emphases depending on the age group in a focus group. The research finding that 6% of babies born to first cousin parents have a genetic anomaly prompted a response from the focus group for older men that this was not very high, *“was it high enough to worry about”* one focus group member asked. These are the sorts of figures that prompted a member of the group made up of older women to attribute the explanation for which child was affected to “ *fate”*. The younger peoples’ focus groups were more accepting of the increased risk being associated with consanguinity. They were more oriented to focus on what steps could be taken to minimise the risk, having an open conversation with the family of the prospective partner and genetic tests before marriage were suggestions made. The salience of genetic risk may vary with the age group of the respondent – for the older groups any genetic anomaly in their family is likely to have already occurred – they may be able to contemplate rates of incidence more dispassionately – if it was going to happen to them it will already have happened. The younger groups engaging with the same level of risk are doing so not knowing what their experience will be, will their child have an anomaly? The salience of maintaining extended family networks and community cohesion may also vary with age. Will a reduction in cousin marriage impact on muti-generational households and will there be a long term impact not just on childcare but also on care of older people?

Members of the older people’s focus groups, particularly the men’s, were wary about information from researchers about cousin marriage and genetic risk. Their concerns were that it was not acknowledging many other possible causes and that, in focussing a discussion of risk on a practice specific to one heritage group, it risked *“demonising”* that group and *“brainwashing”* its children. Participants argued that if cousin marriage and genetic risk was to be discussed it should not be done by schools and, if it was to be done by the medical profession (or researchers), it should be in a *“balanced”* manner. How data is acquired, how it is presented and who presents it are important. There was a suggestion in the younger men’s group that trusted sources were needed to best communicate information, these might be a professionals of South Asian background, or community elders. The extended family could also be a resource. One member of the older women’s group told us she was *“having discussions with her grandchildren”* and advising marrying out of the family.

Our focus groups illustrate the challenge we described in earlier sections, the complexity of multiple causes of genetic anomalies, of risk in all sections of society but increased risk in some, of having risk factors (being married to a cousin for example) but of children not having anomalies. Health education, health promotion and public health messages in this context require nuance and sensitivity. Our focus groups also illustrate the barriers to achieving this sort of response. If a target community feels *“demonised”* via simplistic rhetorical positions taken by politicians, the media, or by some of the professionals they encounter, they are less likely to accept the efforts of those they perceive to be outside their community as accurate and reliable. Over-simplification and vilification are bad for your health. But the focus groups also point to a resolution. Trust is the barrier to acceptance, so seeking and communicating using trusted intermediaries is a way forward. So too is seeing community cohesion as a resource not a barrier. We hear in the focus groups the way older community members can communicate about choice and risk with the younger members “ *WhatsApp Aunties*” to engage with older women who share information via text messages being one example. The belief that a community who seeks to preserve its distinct identity can be a barrier to new ideas or to the acceptance of unwelcome messages must be challenged with a recognition that such communities have effective, within community, networks that, if properly accessed, can be a significant resource for passing on information and offering guidance and support. Extended families and biraderi are important resources for social support. They can be a conduit for effective communication of respectfully fashioned health information.

None of our focus groups spoke specifically about Born in Bradford, or about the many efforts emanating from Bradford health services, local government or the voluntary sector linked to health messages and services related to genetic disorders. There was however an awareness of links between cousin marriage and these disorders that permeated the focus group discussions.

### Some thoughts about theoretical issues

BiB’s reporting significant drops in rates of consanguinity in Bradford provides a context to consider the way a social practice in one community changes over time and to consider how this provides insights into wider social change.

Shenk *et al.* (2024) propose a model of intensive and extensive kinship networks as a means to predict correlates of consanguineous marriage in Pakistan. Intensive kinship networks increase and extensive networks decrease the likelihood of consanguineous marriages. Intensive kinship networks are a characteristic of societies centred on agriculture. The number of cousins in the family, parental consanguinity, spousal proximity, customary caste or clan endogamy, low engagement by women in education and low literacy levels are all linked to high levels of consanguinity. Extensive networks are a feature of societies based on market engagement. They characteristically see greater sustained engagement in education. Here, “a preference for marrying kin is replaced by marriage within your class” ( Shenk *et al.*, 2024). 

The extensive networks Shenk *et al.* describe are reminiscent of what Granovetter (1973) saw when he described the importance of weak ties. This social network theory argues that seemingly weak social connections (even casual acquaintances) can be more valuable than strong ties (close friends and family) in accessing new information and opportunities, particularly in areas like job searching or learning about new ideas. Weak ties can bridge different social circles and provide access to diverse networks. It is an approach to understanding the way social network’s function that has been linked with the development of market economies and with the conditions necessary for a vibrant participatory democracy (see Woodley & Bell, 2013). A study of the detail of patterns of consanguinity and of its resilience in Pakistan therefore offers a route to look at social change at an interface of modernity and tradition.

The literature on acculturation (the process by which people change behaviour, values, and cultural attitudes when they come into contact with another culture) has been widely used to consider social change in migrants and in their descendants Acculturation embraces a spectrum of possible changes from discarding one’s heritage culture to rejecting the receiving culture and retaining the heritage culture, assimilation and separation respectively. There are many possible configurations between these two poles. In our focus groups different sorts of acculturation exist side-by-side. Moves away from consanguineous marriages in the younger members of our Pakistani heritage participants in BiB exist alongside both young and older people seeking to retain heritage aspects of their culture.

The language used in the acculturation literature, national society/receiving society and heritage culture, do not sit comfortably with the longevity of this Pakistani heritage community in Bradford ( Small, 2012). Nor does it sit comfortably with a critical approach to the idea that there is a recognisable and homogeneous national culture that migrants respond to ( Small, 2023, pp131–148). Further, that capacity to engage with both one’s heritage culture and the culture of the surrounding national society is impacted by the discrimination minorities experience. People “approach the national society with the same degree of respect that has been accorded to them” ( Berry *et al.*, 2006). Acculturation is not only something that those arriving have to consider, it also sets a task for the “national” society if they seek to pass on information they hope all will accept.

We have discussed how a shift in patterns of consanguinity may be indicative of changes in the balance between personal choice and traditional practice. This is not classically what we might capture as acculturation. Rather, it is more of a hermetic debate within a culturally distinct sub-group (rather than a debate between one culture and another). In this debate we see the manifestation of a cultural hybridity that captures important areas of the generality of our urban experiences, that is lively “within community” differences while the community as a whole retains a distinct identity (see Werbner & Modood, 1997).

In the Nineteenth Century Tönnies ( 2001: first published 1887) described European modernization as including a shift from community to society (or association) – from relationships based on the family and guild (perhaps in our example biraderi) to relationships based on rationality and calculation. He used the terms gemeinschaft and gesellschaft to capture this. Gemeinschaft describes a world of close, emotional, face to face ties, an attachment to place, to ascribed social status, and to a homogeneous and regulated community. Gesellschaft is about an impersonal urbanism, industrial life, mobility, heterogeneity. Our findings suggest that this is not a distinction that fits well with our Bradford based Pakistani heritage society. There are choices made that are based on rationality and calculation – education, the timing of marriage and of having children, a recognition that some of the traditional links with Pakistan are not relevant to the choices our young people are making (the discussion on land ownership for example). But family, close ties, a respect (albeit a qualified one) for ascribed social status and overall, a sense of a resilient community remain key to their lives. This is an area of classical sociological theory, like the assimilation/acculturation theory, that needs to be revisited in the context of close knit migrant communities living in the UK who experience continuing disadvantage and discrimination. It is a scenario which makes gemeinschaft resilient to a wider societal gesellschaft.

If attitudes to marriage, including a continuing role for consanguinity, gives us an insight into the way modernity and the assumptions of market-economics are mediated by tradition in this community another example is the continuing relevance of biraderi in the politics of the Bradford Pakistani heritage community. Biraderi may influence voting behaviour. It is a form of gemeinschaft, expressed in a political engagement shaped by close ties and is relevant for debates about the importance of weak ties for participatory democracy. It is also, like marriage choice, a domain characterised by differences between community members. Critical voices respond to what is seen by some community members, Akhtar (2013) and Peace and Akhtar (2015) point specifically to the voices of the young, as “the politics of elsewhere” and “of the past”.

This sort of resilient community is also relevant in that part of our data that says economic changes are not significant re changing marital patterns. It may be that the concept of social capital is a more useful one than the narrower economic idea of survival and wealth retention identified in our consideration of the literature on marriage choice. Putnam (2000) says bonding social capital tends to reinforce exclusive identities and homogeneity, bridging social capital tends to bring people together across social divisions ( Putnam, 2000, 22–3). Keeping ones’ peer groups intact as the main sort of financial support is an example of bonding while bridging social capital reaches from one group to another. We see both sorts of social capital coexisting in our Pakistani heritage population (see Bhatia *et al.*, 2024 on biraderi and social capital). One of the ways of reaching beyond one’s own group is by an emphasis on the importance of education and we have seen that this is a key variable in the characteristics of the consanguineous.

Our focus groups also discussed concerns that equating consanguinity with deleterious impacts on children’s health and well-being can be used to stigmatise not just consanguineous families but the wider community where consanguinity is commonplace. This sort of stigmatisation, or even the suspicion that it is taking place, can foster community concerns about how research data will be used and inhibit take up of health education, health promotion and preventative interventions, including genetic counselling and screening services. Having culturally sensitive service provision, in the context of non-stigmatising public discourse, and a target population who are not mistrustful of research means that making informed choices can become the default position. While the link between consanguinity and ill-health is well established researchers and service providers need to build further on work already underway, and cited above, to help people understand the nature of risk in a way that facilitates their making informed choices.

Health literacy rose in importance in the academic literature from the 1970’s in response to interest in the capacities of people to meet the complex demands of health in a modern society ( McQueen *et al.*, 2007). New challenges for achieving health literacy arise from advances in genetics, including those described above, and more generally from advances in personalised medicine. As well as having to understand, appraise and apply health related information there is also a need to understand chance, risk and determinism as well as the conceptual understanding of what genetics is and how its interfaces with context and behaviour in shaping health.

Our focus on consanguinity also underlines other variables, an enhanced risk embedded in the extended family and the impact of the cultural context of a minority group. The factors we have encountered in this study include not just one’s own experience with illness and with the healthcare system but the experience of one’s wider community. Sørensen *et al.* (2012) pointed to the importance of including in health literacy an appreciation of proximal and distal factors that impact on health. Distal included demographic, psychosocial and cultural factors and proximal factors included general literacy as well as individual characteristics including prior experience with illness and with the healthcare system. In this conceptualisation of health literacy, it is not just people being able to understand what health professionals say but also health professional having to adapt their message so that there pronouncements can be understood. They need to develop a communicative literacy that includes a sensitivity to all the factors impacting on all the parties. Achieving health literacy then involves a two way interaction and a mutual willingness to adapt one’s position. The gains can be considerable.

There are individual, interactive and community benefits of health literacy. Individual benefits include improved knowledge of risks and of services and increases in compliance with prescribed actions. Interactive health literacy sees an improved capacity to act independently, to have better motivation, self-confidence, and a resilience to adversity. Community and social benefits move us beyond the individual to see health literacy leading to increased participation in population health programmes, changes in social norms and enhanced community empowerment, including in the ability to act in relation to social and economic determinates of health ( Nutbeam, 2000; Nutbeam, 2008). Health literacy is a personal and communal asset. Kickbusch and Maag (2008) identified that “a person with an adequate level of health literacy has the ability to take responsibility for one’s health and family and community health”.

### Shortcomings

The reduction in rates of cousin marriage we used as a motivation to set up this qualitative study collected data up to 2019. Our focus group members thought rates now were (much) lower, Small *et al.*, 2024a hypothesised that figures showing decline in cousin marriages (from being a majority to a minority in just 9 years) was part of a trend, a downward trajectory that might accelerate as cousin marriage became at odds with the peer group norms of younger people. In consequence our data from Small *et al.* (2024a) and the focus group data we present here are not synchronised.

The focus group study is small and from one city. We have emphasised the importance of local factors shaping community practices above but it is also of note how concerns about changing patterns of consanguinity and about health and well-being implications for the offspring of such unions are being considered in many countries where consanguinity is common (we have cited research focussed on Pakistan, Qatar and South India as examples above).

Our decision to set up groups for different age groups and genders meant we did not have the opportunity for discussion between them that might have illuminated the degree and resonance of the differences we report.

BiB is seeking to develop an on-line qualitative data repository and also make qualitative data available via other data repositories. This was not possible with data from this study but, in future, we will adapt our ethics submissions and consent procedures so that when such a resource is available we can share data. Quantitative data from BiB is available (details below).

### A self-reflection

The research team includes members of the Pakistani heritage community and researchers with long-experience of the dynamics of this community. We considered how far the focus group discussions were consistent with our experiences/knowledge of this community. We have commented above on how we found group members willingly engaged with the questions we asked and provided the rich insights we report. Our team, consistent with the views of focus group members, thought the proportion of cousin marriages in this community had decreased considerably since the data Small *et al.* (2024a) report.

For example, team members report:

*“The last few weddings I have attended have been between people who are not only not related to each other but also happen to have family heritage from different villages and therefore unlikely to have had any previous liaisons with each other. Similarly, when I reflect on the weddings of many of my friends and relatives which happened in the last decade or so, then majority of them were between people who were unrelated. I can’t remember when I last went to a marriage between cousins; and for that reason, I expected the numbers to be much lower. ”*

“ *It was interesting for me to note younger people all felt it was important they married someone who they shared an ethnic or cultural identity with. It wasn’t a case of they wanted to abandon the notion of culture, lock stock and barrel, but it was more a case of culture encompassed a bigger circle than simply their parents’ language, birth village and relations. But this new latitude came with new boundaries; marrying someone from, say a non-South Asian background, was not included in this circle. There is a tacit agreement at play which makes it important that the potential spouse must be close to the cultural boundary (whatever such a boundary may entail) even though that has widened to include people who may have been off limits in the not-so-distant past.”*

Team members also shared with some focus group members a sense of continuing social cohesion despite the generational differences our focus groups report:

*“Through reading the transcripts I could sense and hear the generational differences between the parents and their young adult children. … It would be a mistake to assume these generational differences are rebellious and hostile; they are not, these are cultural changes, which both generations see as inevitable.”*

## Conclusion

Our focus groups enabled us to explore why people think changes in patterns of marriage choice are occurring and to reflect on their significance. They also allow us to consider how far a consideration of enhanced health risk in children feature in the social changes our data report.

Older people were concerned about a reduction in consanguinity signifying a shift from traditions that were important for both community identity and solidarity. They regretted witnessing the demise of their family collectivist culture as it moved towards a more individualist one. The younger people saw the new social milieu as positive and they valued the opportunity to marry a partner for reasons other than family ties and tradition. They wanted to be able to make individual choice but did not want to lose other aspects of their culture that they valued, including a role for families in supporting them in their marriages. There was less sense of the importance of continuing links with Pakistan in our younger respondents.

There was knowledge about health risk but some concern that the explanation was more complex than a simple attribution to consanguinity. There were a number of participants in the older men’s group who went further and contested the link between cousin marriage and congenital anomalies. The way messages about risk were communicated was criticised and the danger of a health risk being subsumed into a cultural practice and used to *“demonise”* those who marry cousins was highlighted. The older men’s group felt this was an attempt to pave the way for restrictions and, in that, was another stick to beat their community with. These reactions indicate how a sense of being in a minority that has experienced discrimination creates barriers to clear communication about health risk. These are reactions that provide an example of how discrimination is bad for your health.

Looking at marriage as both an intimate choice and a well-established community practice allow us to explore how modernity is being shaped in a long-established migrant community. It prompts a critique of aspects of prevailing theories and reminds us that disadvantaged communities who also experience discrimination balance individual choice and community cohesion in ways that differ to those in their “host” communities.

We are speaking about something, “cousin marriage”, that is specific to one community – but the more general concerns being expressed and the differences between older and younger members of the Pakistani heritage community are much more commonplace across all communities – for example are changing times to be welcomed or should we be anxious about what changes will do to those things we want to preserve?

## Ethics and consent

Approval for Born in Bradford was provided by Bradford Local Research Ethics committee (reference number 07/H1302/112 – approval date 1/4/2008). Ethical approval for the Born in Bradford Better Start study was given by Bradford Leeds NHS Research Ethics Committee (15/YH/0455) on 10/11/2015.

Research governance approval has been provided from Bradford Teaching Hospitals NHS Foundation Trust. All study participants in Born in Bradford were given Participant Information Sheets approved by the Ethics Committee before recruitment and all participants signed consent forms which included consent for data collection, usage, and data sharing.

The focus group aspects of this study were approved in a separate research ethics review by the Research Ethics Panel at the University of Bradford on 31 August 2023. The reference identity number is E1112.

Written consent was obtained from all focus group participants after they had been provided with a comprehensive information sheet about the study and been given an opportunity to ask any questions. Both the information sheet and consent form are provided in our Extended Data.

## Data Availability

The transcripts of focus groups reported in this study are not available for access. Rather, we present anonymised extracts of data with only the identification of the focus group the contribution was made in included. Focus group discussions included much detail about individuals lives and about their families and neighbours. Further, all participants were from one geographical area. If full transcripts were made available, even after being anonymised, it might be possible to identify individual contributors by piecing together biographical details. A condition of ethical approval for the focus group data reported was that tape recording of the groups would be transcribed and that the tapes and transcripts would be saved on a secure NHS system for the research team to access. They would then analyse, anonymise and present summaries of the discussions. All focus group material would be kept for eighteen months and then destroyed. There is a wide-ranging data set available from BiB and BiBBS. This is data that underpins the study we report in this paper. Researchers are encouraged to make use of this data, which are available through a system of managed open access. Before you contact us, please make sure you have read our Guidance for Collaborators. Our BiB Executive reviews proposals on a monthly basis and we will endeavour to respond to your request as soon as possible. You can find out about the different datasets in our Data Dictionary. If you are unsure if we have the data that you need, please contact a member of the BiB team ( borninbradford@bthft.nhs.uk). Once you have formulated your request please complete the ‘Expression of Interest’ form available here and send to borninbradford@bthft.nhs.uk. If your request is approved we will ask you to sign a Data Sharing Contract and a Data Sharing Agreement, and if your request involves biological samples we will ask you to complete a material transfer agreement. Data are available under the terms of the Creative Commons Zero "No rights reserved" data waiver (CC0 1.0 Public domain dedication). We have made available the information sheet participants were given; the consent form they signed and the questions used to begin discussion in the focus groups. In addition, we have made available a completed Standards for Reporting Qualitative Research (SRQR) form which is a widely used tool for linking understandings of academic rigour in qualitative research with specific aspects of this paper. Harvard Dataverse: Replication Data for: Changing patterns in marriage choice and related health risk in the Pakistani heritage community in Bradford UK: a qualitative study. Extended Data. https://doi.org/10.7910/DVN/PVK9KW ( Small *et al*., 2024) This project contains the following extended data: Focus Group Information Sheet Focus Group Consent Form Focus Group Topic Guide/questions. Standards for Reporting Qualitative Research (SRQR) completed form. Data are available under the terms of the Creative Commons Zero "No rights reserved" data waiver (CC0 1.0 Public domain dedication).
