# Supplementary material for: Changing patterns in marriage choice and related health risk in the Pakistani heritage community in Bradford UK: a qualitative study
Source: Wellcome Open Res. 2025 Jun 9;9:690. Originally published 2024 Nov 22. [Version 3] doi: 10.12688/wellcomeopenres.23338.3 (PMC12120418; doi:10.12688/wellcomeopenres.23338.3)
Supplement: Peer review history

# Reviewer report: Reviewer response for version 3

**DOI:** 10.21956/wellcomeopenres.26891.r125338

**Published:** 2025-06-16

**Authors:** Saeed Anwar

**Affiliations:** 1 University of Alberta, Edmonton, Canada

The authors have addressed the majority of previous concerns with care and thoughtfulness. Clarifications on recruitment strategy, group composition, and thematic analysis have improved transparency. I also appreciate the authors for acknowledging potential sampling biases, particularly the underrepresentation of culturally distanced individuals. The use of acculturation and health literacy frameworks improves the manuscript's sensitivity, though these theoretical elements are somewhat basic.

 A clearer discussion of the researcher’s perspective would enhance transparency but does not detract from the study's value. The overall findings offer valuable insight into views on consanguinity within a specific diasporic context and can inform community health dialogues and policy.

 I encourage the authors to continue developing reflexive and theoretical depth in future work to complement their community-centered research approach.

Is the work clearly and accurately presented and does it cite the current literature?

Partly

If applicable, is the statistical analysis and its interpretation appropriate?

Not applicable

Are all the source data underlying the results available to ensure full reproducibility?

Partly

Is the study design appropriate and is the work technically sound?

Partly

Are the conclusions drawn adequately supported by the results?

Partly

Are sufficient details of methods and analysis provided to allow replication by others?

Partly

Reviewer Expertise:

Medical genetics, community genetics, genetic therapeutics, epidemiology, health disparties.

I confirm that I have read this submission and believe that I have an appropriate level of expertise to confirm that it is of an acceptable scientific standard.

# Reviewer report: Reviewer response for version 3

**DOI:** 10.21956/wellcomeopenres.26891.r123367

**Published:** 2025-06-14

**Authors:** Atta Ur Rehman

**Affiliations:** 1 Hazara University, Mansehra, Khyber Pakhtunkhwa, Pakistan

The manuscript describes a shift in marriage preferences among the Pakistani community living in the UK and explains the possible reasons and outcomes of this transition especially in terms of health risk in future. I believe that the study is scientifically sound, study approach is justified, and the results are sufficiently supported by relevant data. Since most, if not all, participants are of Pakistani descent, I would suggest that the reviewers could improve background as well as discussion of this study by comparing and contrasting previous studies conducted in Pakistan. This is of particular importance because consanguinity have been varied across different ethnic or geographic localities in Pakistan. The authors might better explain, discuss or contextualized their findings by reading/citing the following articles: ( refer to 1, 2 , 3 and 4)

Is the work clearly and accurately presented and does it cite the current literature?

Partly

If applicable, is the statistical analysis and its interpretation appropriate?

I cannot comment. A qualified statistician is required.

Are all the source data underlying the results available to ensure full reproducibility?

Yes

Is the study design appropriate and is the work technically sound?

Yes

Are the conclusions drawn adequately supported by the results?

Yes

Are sufficient details of methods and analysis provided to allow replication by others?

Yes

Reviewer Expertise:

Human Genetics, Genetic Epidemiology, Public Health, Genomic Medicine

I confirm that I have read this submission and believe that I have an appropriate level of expertise to confirm that it is of an acceptable scientific standard.

## References

[1] : Consanguinity in northwest Pakistan: evidence of temporal decline. *Journal of Biosocial Science* .2024;56(3) : 10.1017/S0021932024000026 445-458 10.1017/S0021932024000026 38314634

[2] https://pubmed.ncbi.nlm.nih.gov/27252925/.

[3] : Determinants of consanguinity and inbreeding coefficient in the multiethnic population of Mardan, Khyber Pakhtunkhwa, Pakistan. *Asian Biomedicine* .2017;11(6) : 10.1515/abm-2018-0022 451-460 10.1515/abm-2018-0022

[4] PMID: 26075334.

# Reviewer report: Reviewer response for version 2

**DOI:** 10.21956/wellcomeopenres.26476.r121456

**Published:** 2025-05-29

**Authors:** Mir Azad Kalam

**Affiliations:** 1 Narasinha Dutt College, Howrah, West Bengal, India

The manuscript is commendably well-written, with particular strength in the methodology section. The authors deserve full credit for their thoughtful and detailed approach, incorporating a range of methodological tools and emphasizing qualitative data to explore the topic of consanguinity. This focus on qualitative aspects—especially intergenerational perspectives—is both rare and valuable, given the limited number of studies addressing this dimension of consanguineous marriage.

 After a thorough and repeated reading of the manuscript, I have a few observations and suggestions for improvement, which are outlined below:

1. **Introduction**: The introduction currently reads more like a general review of consanguineous marriage. In my opinion, there is no need to elaborate extensively on definitions, causative factors, or general background unless directly relevant to the study. If such information is essential, I suggest condensing it to a more concise form to maintain focus and avoid redundancy.
2. **Methodology**: While the methodology section is robust, one point requires clarification. It is not clear whether the young male and female participants were married or unmarried at the time of the interviews. Please specify this, as it may influence their perspectives on consanguinity.
3. **Participant Selection**: Apart from the Born in Bradford (BiB) study cohort, were there any other inclusion or exclusion criteria for selecting participants? Additionally, it would be helpful to clarify whether the older male and female participants were also born in Bradford, as this may have implications for interpreting generational differences. If possible, mention the background socio-economic characteristics in the method section.
4. **Quantitative Data**: While the study is primarily qualitative, the inclusion of some basic statistical or demographic data would help contextualize the findings. For instance, presenting trends or changes in consanguinity rates over time, especially within the Bradford area, would strengthen the discussion and provide a clearer picture of the shifting patterns.

Overall, this is an insightful and much-needed contribution to the literature on consanguinity, and I look forward to seeing the revised version.

Is the work clearly and accurately presented and does it cite the current literature?

Yes

If applicable, is the statistical analysis and its interpretation appropriate?

Partly

Are all the source data underlying the results available to ensure full reproducibility?

Yes

Is the study design appropriate and is the work technically sound?

Yes

Are the conclusions drawn adequately supported by the results?

Yes

Are sufficient details of methods and analysis provided to allow replication by others?

Yes

Reviewer Expertise:

ANTHROPOLOGICAL DEMOGRAPHY, CONSANGUINITY, MIGRATION

I confirm that I have read this submission and believe that I have an appropriate level of expertise to confirm that it is of an acceptable scientific standard.

## References

[1] : Linkages between consanguinity, pregnancy outcomes and offspring mortality in twenty-first century India. *Sci Rep* .2024;14(1) : 10.1038/s41598-024-69151-7 22522 10.1038/s41598-024-69151-7 39341841 PMC11438857

# Response

**Published:** 2025-06-04

**Authors:** Neil Small

**Affiliations:** Faculty of Health Studies, University of Bradford, Bradford, England, UK

*We are grateful for this reviewers’ supportive comments. We are also grateful for the suggested changes that could improve our article. We have responded to each point below.  *

1. **Introduction**: The introduction currently reads more like a general review of consanguineous marriage. In my opinion, there is no need to elaborate extensively on definitions, causative factors, or general background unless directly relevant to the study. If such information is essential, I suggest condensing it to a more concise form to maintain focus and avoid redundancy.

*We see the point the reviewer is making. But, as we have discussed in the paper, this qualitative paper links with two papers published in Wellcome Open shortly before this paper was submitted (Small et al 2024a and Small et al 2024b.)   These looked at morbidity in children and at changing rates of consanguinity. Our qualitative study was designed to investigate the empirical findings from these papers – we developed our study protocol to do this. But we also wanted to look at the literature on health risk and reasons for choosing cousin marriage and shape our study so that our empirical data would critique and complement this scholarship. The detailed picture we offer is intended to make these antecedents available to readers of this article. .  *

1. **Methodology**: While the methodology section is robust, one point requires clarification. It is not clear whether the young male and female participants were married or unmarried at the time of the interviews. Please specify this, as it may influence their perspectives on consanguinity.

*We are grateful for this point – we have added this detail in our Methods section. None of our younger male and female participants were married (marriage status was not one of inclusion criteria so this was not by design.)*

1. **Participant Selection**: Apart from the Born in Bradford (BiB) study cohort, were there any other inclusion or exclusion criteria for selecting participants? Additionally, it would be helpful to clarify whether the older male and female participants were also born in Bradford, as this may have implications for interpreting generational differences. If possible, mention the background socio-economic characteristics in the method section.

*The criteria were that people live in Bradford, that they were of Pakistani heritage, that they fell into the broad age groups we were targeting and that they consented to take part.* *Re: were older group members born in Bradford – in the older men’s group 2 were born in Pakistan the rest were born in Bradford. In the older women’s group one was born in Pakistan and the others were born in the UK.* *Re: socio-economic characteristics – in the older groups there was a mixture of self-employed and employed alongside homemakers and not-employed. We did not ask for these details so this is what we can infer from what people said in our focus groups. Likewise for current marital status – all older group members were or had been married.* *We have added these details in our “Methods” section.  *

1. **Quantitative Data**: While the study is primarily qualitative, the inclusion of some basic statistical or demographic data would help contextualize the findings. For instance, presenting trends or changes in consanguinity rates over time, especially within the Bradford area, would strengthen the discussion and provide a clearer picture of the shifting patterns.

*We appreciate that some basic statistical and demographic data is likely to be of interest to our readers. We have not provided this because we wanted to keep the focus on our qualitative data and also because we have published a considerable amount of such detail in the papers that inform it. In our “Background” section we do mention that high levels of deprivation and the ethnic mix in the city. We also report changing rates of consanguinity between our two cohort recruitment periods.  But we do no present great detail re demographics. We do cite two detailed papers on cohort characteristics (Wright et al 2013 and Dickerson et al 2022) which include very detailed demographics and our papers on morbidity and on changing rates of consanguinity during our recruitment periods which also have much contextual detail (Small et al 2024b and Small et al. 2024a). We appreciate that our making this decision will make it a little more demanding for our readers who want just to access this specific article, they will need to follow the links in our citations to gather statistical and demographic detail.*

# Reviewer report: Reviewer response for version 2

**DOI:** 10.21956/wellcomeopenres.26476.r121350

**Published:** 2025-04-09

**Authors:** Saeed Anwar

**Affiliations:** 1 University of Alberta, Edmonton, Canada

The authors have addressed several of this reviewer’s critiques. They have clarified key aspects of recruitment, sampling, and thematic analysis, including participant inclusion criteria and the rationale for age and gender group divisions. The manuscript now engages more meaningfully with models of acculturation and health literacy, and the limitations section has been improved to acknowledge recruitment bias and the potential exclusion of more acculturated individuals. These revisions are appreciated and contribute positively to the manuscript’s transparency. However, some areas still require further refinement. Some characterizations risk subtly reinforcing stereotypes.

 The portrayal of older participants as tradition bearers and younger individuals as drivers of change may essentialize generational roles and oversimplify complex identity negotiations. Additionally, the suggestion that more acculturated individuals have “drifted away” from community strengths appears to uncritically valorize tradition. Discussions of “control” (e.g., managing a wayward son through cousin marriage) require more careful contextualization to avoid perpetuating negative perceptions. Such narratives should be framed critically and supported with theoretical reflection, not anecdotal emphasis. While the authors acknowledge recruitment bias, there seems to be limited triangulation of findings. Although further data collection may not be feasible, the study’s constraints, particularly the limited representativeness of culturally distanced voices, should be emphasized more prominently in both the abstract and conclusion. The incorporation of acculturation and health literacy frameworks is a step forward but remains introductory. A deeper engagement with constructs, e.g., bicultural identity, intersectionality, and structural barriers, would enhance the theoretical richness of the analysis. The positionality of the research team, particularly the facilitators and analysts, could be clearer.

 A reflexive statement addressing their relationship to the community, cultural fluency, and influence on the data collection and interpretation process would strengthen the manuscript’s transparency and trustworthiness. The use of the term “fallback plan” regarding consanguinity may be perceived as pejorative and should be handled with greater care. It is better to avoid directly framing traditional marriage choices as risky or undesirable.

 Lastly, while the organization of themes has improved, a tendency toward overgeneralization from a limited dataset still remains. Some conclusions, especially those linking immigration law to consanguinity trends, are speculative and require stronger empirical grounding.

Is the work clearly and accurately presented and does it cite the current literature?

Partly

If applicable, is the statistical analysis and its interpretation appropriate?

Not applicable

Are all the source data underlying the results available to ensure full reproducibility?

Partly

Is the study design appropriate and is the work technically sound?

Partly

Are the conclusions drawn adequately supported by the results?

Partly

Are sufficient details of methods and analysis provided to allow replication by others?

Partly

Reviewer Expertise:

Medical genetics, community genetics, genetic therapeutics, epidemiology, health disparties.

I confirm that I have read this submission and believe that I have an appropriate level of expertise to confirm that it is of an acceptable scientific standard, however I have significant reservations, as outlined above.

# Reviewer report: Reviewer response for version 2

**DOI:** 10.21956/wellcomeopenres.26476.r121349

**Published:** 2025-04-04

**Authors:** Theresa Thompson Chaudhry

**Affiliations:** 1 Lahore School of Economics, Burki, Pakistan

I am satisfied with the revisions.

Is the work clearly and accurately presented and does it cite the current literature?

Partly

If applicable, is the statistical analysis and its interpretation appropriate?

Not applicable

Are all the source data underlying the results available to ensure full reproducibility?

No

Is the study design appropriate and is the work technically sound?

Partly

Are the conclusions drawn adequately supported by the results?

Yes

Are sufficient details of methods and analysis provided to allow replication by others?

No

Reviewer Expertise:

applied microeconomics, empirical methods

I confirm that I have read this submission and believe that I have an appropriate level of expertise to confirm that it is of an acceptable scientific standard.

# Reviewer report: Reviewer response for version 1

**DOI:** 10.21956/wellcomeopenres.25722.r113172

**Published:** 2024-12-16

**Authors:** Theresa Thompson Chaudhry

**Affiliations:** 1 Lahore School of Economics, Burki, Pakistan

The paper is a worthy contribution to the literature if some adjustments are made to the discussions of the literature and the methodology is more carefully explained.

 On pg. 4-5, the discussion of the increased risk due to consanguinity was incomplete. One of the studies cited in a paragraph intended to demonstrate the risk, Mobarak et al. (2019), has been misrepresented. They actually find that the impact of consanguinity on mortality and morbidity was statistically insignificant once they accounted for: (a) non-random selection into consanguinity, and (b) correlations in health outcomes across offspring of the same union. Further, Mobarak et al. (2019) suggests that the results of Sheridan et al. (2013) should be re-considered in light of these issues. Bittles & Black (2010) also hypothesize that once selection into consanguinity and population sub-structure are properly accounted for, “the mean value of 3.5% excess mortality at the first-cousin level is an upper-level estimate that may be subject to further downward revision as data from better-designed studies become available.”

1. Bittles, Alan H., and Michael L. Black. "Consanguinity, human evolution, and complex diseases." *Proceedings of the National Academy of Sciences* 107.suppl_1 (2010): 1779-1786.

In addition to the changes to Immigration Law impacting the availability of cousins to marry, falling fertility rates should also be considered a factor. See:

1. Barakat, Bilal, and Stuart Basten. 2014. "Modelling the Constraints on Consanguineous Marriage When Fertility Declines." Demographic Research 30: 277-312.
2. Shenk, M.K., Naz, S. and Chaudhry, T. (2024), Intensive Kinship, Development, and Demography: Why Pakistan has the Highest Rates of Cousin Marriage in the World. Population and Development Review. https://doi.org/10.1111/padr.12678

The discussion of bonding and bridging social capital on pg. 18 would be bolstered by a reference to the work of Shenk et al. (2024), which frames different modes of marriage as intensive or extensive kinship.

 The methodology seemed opaque to me. But as an economist (who mainly does quantitative  work), I am less comfortable commenting in detail on the qualitative methods employed. I would agree, however, with the other reviewer who suggested that fluency in English might have been a constraint. I found the quotations from the sub-samples of mature/older individuals challenging to comprehend, and a reason for that may have been language problems.

**Minor comments:**

 Word choice, on pg 4:

 “To examine how **far** health and education outcomes linked to consanguinity are known to the Pakistani heritage community…” could be more easily understood if phrased as “To examine **to what extent** health and education outcomes…”

 There is a confusing passage, top of pg. 6, where it says, “It may be that we are seeing in the figures we present above a shift presaged by Shaw nearly 20 years before but moving beyond the cousin marriage that was still dominant then. Studying consanguinity may indicate a more prevalent affective individualism shaping a different sense of self and society…”

 Comments: First, there is no figure above. Second, what shift was foreseen by Shaw? It has not been discussed.

 Possible incomplete sentence on pg. 6, where it says, “If cultural and faith-based concerns are important then the congruence of attitudes between the two settings is important and if they are diverging that will impact ___.”

 There were some misplaced apostrophes in a couple of the quotes of participants:

 pg. 14: “The biggest thing in our family was when one of my **cousin’s** married his second cousin…”

 pg. 14: “And then my dad’s elder brother and his wife, again, **we’re **first cousins...”

Is the work clearly and accurately presented and does it cite the current literature?

Partly

If applicable, is the statistical analysis and its interpretation appropriate?

Not applicable

Are all the source data underlying the results available to ensure full reproducibility?

No

Is the study design appropriate and is the work technically sound?

Partly

Are the conclusions drawn adequately supported by the results?

Yes

Are sufficient details of methods and analysis provided to allow replication by others?

No

Reviewer Expertise:

applied microeconomics, empirical methods

I confirm that I have read this submission and believe that I have an appropriate level of expertise to confirm that it is of an acceptable scientific standard, however I have significant reservations, as outlined above.

# Response

**Published:** 2025-03-17

**Authors:** Neil Small

**Affiliations:** Faculty of Health Studies, University of Bradford, Bradford, England, UK

The paper is a worthy contribution to the literature if some adjustments are made to the discussions of the literature and the methodology is more carefully explained.

 On pg. 4-5, the discussion of the increased risk due to consanguinity was incomplete. One of the studies cited in a paragraph intended to demonstrate the risk, Mobarak et al. (2019), has been misrepresented. They actually find that the impact of consanguinity on mortality and morbidity was statistically insignificant once they accounted for: (a) non-random selection into consanguinity, and (b) correlations in health outcomes across offspring of the same union. Further, Mobarak et al. (2019) suggests that the results of Sheridan et al. (2013) should be re-considered in light of these issues. Bittles & Black (2010) also hypothesize that once selection into consanguinity and population sub-structure are properly accounted for, “the mean value of 3.5% excess mortality at the first-cousin level is an upper-level estimate that may be subject to further downward revision as data from better-designed studies become available.” *We are grateful for this comment and have changed the text of our article accordingly. We agree that the text of our original article misrepresented Mobarak et al (2019) by not reporting their findings re non-random selection and correlations across offspring from the same union. We have added more detail on Mobarak et al in our revised text. We have also added a short section on endogamy with specific reference to biraderi.* *We have looked at the characteristics of those of Pakistani heritage in the Born in Bradford cohort and compared these for consanguineous and non-consanguineous couples. There are differences but none reach significance so that they suggest factors that might influence health-outcomes in offspring that are the equivalent or near equivalent of consanguinity (Sheridan et al. 2013  Bhopal  et al 2014). There are also differences in consanguinity rates according to biraderi population sub-structures (themselves linked to historic social positions/occupations.) This does suggest non-random selection – there are many other factors that might be significant in selection - family history, migrations status – but because of the wide range of possibilities these may be best explored in qualitative studies. We did not include such questions in this study where a main focus was the reduction in rates of consanguinity across our two cohort recruitment periods and where we had identified demographic characteristics that were linked to it (age/education/migration history). Further, it is likely that potential co-factors such as non-random selection were similar across our two time periods because the population our data was drawn on was very similar. .* *We have not looked at health outcomes across offspring of the same union. There were couples in the Born in Bradford study  who had more than one child during the years the study recruited participants but their numbers were too small to allow meaningful analysis.* *Re the Bittles and Black (2010) paper – we hope that the Sheridan et al study and subsequent work from Born in Bradford cited in our article means that our study may be seen as providing data from a “better designed study” That Bittles and Black were hoping for. In a Comment published in The Lancet after the publication of Sheridan et al Bittles said this article was “a welcome and important source of detailed information against which some of the pros and cons of intrafamilial marriage within contemporary communities can be assessed objectively” Bittles, A H 2013. “Consanguineous marriages and congenital anomalies”* *www.thelancet.com* *Vol 382 Oct 19. Bittles did express regret that biraderi was not included in Sheridan et al and he has worked subsequently with the Born in Bradford team to explore that dimension of the Bradford experience – work with have cited in our paper (see Bittles  and Small 2016 and Small, Bittles, et al 2017 )*      In addition to the changes to Immigration Law impacting the availability of cousins to marry, falling fertility rates should also be considered a factor. 

*We are grateful for this point. In the two recruitment periods for the studies reported in our article we did not note falling fertility rates in Bradford. The time period between the studies (9 years) may be too short to see such changes. We did find fertility rates to be higher in the Pakistani heritage population compared with the White British population across the whole time period of recruitment to the Born in Bradford studies. We do take the point that a change in fertility rates in Pakistan (and specifically in Azad Kashmir) might reduce the numbers who might be seen as potential marriage partners. We have added a note to link with our point about changing Immigration Laws to reflect the potential for fertility to play a part in reductions in consanguinity. We now include the Barakat et al paper in our references. *   The discussion of bonding and bridging social capital on pg. 18 would be bolstered by a reference to the work of Shenk et al. (2024), which frames different modes of marriage as intensive or extensive kinship.

*We had not seen the Shenk et al article before submitting our paper (it was published in November 2024) and we are grateful to have it pointed out to us. It is a most interesting contribution to a literature that we consider very important. We have added a note to underline the value of Shenk et al to our study.  *   The methodology seemed opaque to me. But as an economist (who mainly does quantitative  work), I am less comfortable commenting in detail on the qualitative methods employed. I would agree, however, with the other reviewer who suggested that fluency in English might have been a constraint. I found the quotations from the sub-samples of mature/older individuals challenging to comprehend, and a reason for that may have been language problems.

*We have now revised the methodology section to provide an in-depth account of the steps involved in the thematic analysis process which will add to the clarity and  replicability of the methods.* *Re fluency in English for our focus group members: the members of our team who undertook the focus groups were fluent in the languages spoken amongst the participants of the groups. We do not think that fluency in English put people off participating and we don’t think we had an unrepresentative sample. But it may be the case that members of the groups of older participants are less fluent in English and that comes over in verbatim quotes. We have cut/shortened quotes and, hopefully, that helps understanding. It is a dilemma many qualitative researchers encounter – should we modify the actual language and the expressions used to aid comprehension. We decided not to do this and we accept this sometimes means trading easier intelligibility for authenticity.*

**Minor comments:**

 Word choice, on pg 4:

 “To examine how **far** health and education outcomes linked to consanguinity are known to the Pakistani heritage community…” could be more easily understood if phrased as “To examine **to what extent** health and education outcomes…” *Thank you, we have changed the text as you suggest.*

 There is a confusing passage, top of pg. 6, where it says, “It may be that we are seeing in the figures we present above a shift presaged by Shaw nearly 20 years before but moving beyond the cousin marriage that was still dominant then. Studying consanguinity may indicate a more prevalent affective individualism shaping a different sense of self and society…”

 Comments: First, there is no figure above. Second, what shift was foreseen by Shaw? It has not been discussed. *We have modified this section to address the concerns expressed and , hopefully, make our points clearer.*

 Possible incomplete sentence on pg. 6, where it says, “If cultural and faith-based concerns are important then the congruence of attitudes between the two settings is important and if they are diverging that will impact ___.”

 There were some misplaced apostrophes in a couple of the quotes of participants:

 pg. 14: “The biggest thing in our family was when one of my **cousin’s** married his second cousin…”

 pg. 14: “And then my dad’s elder brother and his wife, again, **we’re **first cousins...” *We have corrected the three mistakes identified.*

# Reviewer report: Reviewer response for version 1

**DOI:** 10.21956/wellcomeopenres.25722.r113173

**Published:** 2024-12-10

**Authors:** Saeed Anwar

**Affiliations:** 1 University of Alberta, Edmonton, Canada

The manuscript in discussion examined cultural shifts in consanguinity and related health risks within Bradford's Pakistani heritage community, using four focus groups stratified by age and gender. It identifies three key themes: the influence of religion, reliance on tradition, and the perceived risks of consanguineous marriages. Generational differences emerge, with younger participants favouring individual partner choice while older participants emphasize family cohesion and tradition. Additional factors discussed include health risks, immigration policies, and societal changes.

 Participants were recruited through mosques, youth clubs, and voluntary organizations, but the authors do not specify if a specific sampling technique was used (e.g., snowball sampling). There is a lack of detail on inclusion/exclusion criteria and demographic diversity, which, along with a small sample size (28–32 participants), raises concerns about the robustness of the conclusions, especially regarding generational and gender differences. The authors should clarify their sampling strategy and justify the sample size, including whether theme saturation was achieved.

 The recruitment strategy may have unintentionally excluded community segments distanced from their original culture due to acculturation and socioeconomic changes. Focusing on culturally specific institutions likely attracted participants connected to traditional networks, leaving less engaged individuals underrepresented.

 Focus groups were conducted in English, potentially excluding less fluent participants and limiting the diversity of perspectives. This linguistic barrier should be acknowledged, and efforts to mitigate its impact should be described.

 The authors mention Braun and Clarke's analysis framework, but the manuscript lacks key details, e.g., coding processes, validation methods (for instance, inter-coder reliability), and an audit trail for theme emergence. The use of subjective saliency criteria for prioritizing themes needs more justification. Including the focus group guide or discussion prompts as supplementary material would improve transparency and replicability.

 The introduction emphasizes the importance of consanguinity but relies too much on past descriptive statistics. A clearer research objective would enhance focus. It also fails to engage with broader theories, e.g., acculturation or health literacy models. Claims linking the decline in consanguinity to education and immigration policies are speculative and need stronger evidence.

 The study captures diverse perspectives, but its conclusions are occasionally overgeneralized. Anecdotal health risks are discussed without considering other confounding factors, and claims about declining consanguinity due to education and policy changes require more cautious framing and empirical support.

 Long, repetitive quotes weaken the findings. Condensed excerpts, clearer theme synthesis, and visual aids like thematic maps could enhance clarity and engage readers more effectively. Can the authors provide an account for this?

 The authors might consider posting raw data, like excerpts or summaries, for transparency and meta-analysis.

 Overall, the study tried to tackle a sensitive topic using a qualitative approach, but methodological flaws, limited theoretical engagement, and speculative interpretations weaken its scientific rigour. Addressing these issues could improve its transparency, validity, and impact.

Is the work clearly and accurately presented and does it cite the current literature?

Partly

If applicable, is the statistical analysis and its interpretation appropriate?

Not applicable

Are all the source data underlying the results available to ensure full reproducibility?

Partly

Is the study design appropriate and is the work technically sound?

Partly

Are the conclusions drawn adequately supported by the results?

Partly

Are sufficient details of methods and analysis provided to allow replication by others?

Partly

Reviewer Expertise:

Medical genetics, community genetics, genetic therapeutics, epidemiology, health disparties.

I confirm that I have read this submission and believe that I have an appropriate level of expertise to state that I do not consider it to be of an acceptable scientific standard, for reasons outlined above.

# Response

**Published:** 2025-03-17

**Authors:** Neil Small

**Affiliations:** Faculty of Health Studies, University of Bradford, Bradford, England, UK

The manuscript in discussion examined cultural shifts in consanguinity and related health risks within Bradford's Pakistani heritage community, using four focus groups stratified by age and gender. It identifies three key themes: the influence of religion, reliance on tradition, and the perceived risks of consanguineous marriages. Generational differences emerge, with younger participants favouring individual partner choice while older participants emphasize family cohesion and tradition. Additional factors discussed include health risks, immigration policies, and societal changes. Participants were recruited through mosques, youth clubs, and voluntary organizations, but the authors do not specify if a specific sampling technique was used (e.g., snowball sampling). There is a lack of detail on inclusion/exclusion criteria and demographic diversity, which, along with a small sample size (28–32 participants), raises concerns about the robustness of the conclusions, especially regarding generational and gender differences. The authors should clarify their sampling strategy and justify the sample size, including whether theme saturation was achieved.

 The recruitment strategy may have unintentionally excluded community segments distanced from their original culture due to acculturation and socioeconomic changes. Focusing on culturally specific institutions likely attracted participants connected to traditional networks, leaving less engaged individuals underrepresented.

*We recognise the importance of being clear about our recruitment strategy – we think the range of sites for recruitment are broad and are likely to have made the study available to people with different interests and backgrounds. But it is true that we were less-likely to recruit people distanced from their original community, particularly via our recruitment through mosques.  But there is a high rate of attendance at mosques and the range of voluntary organisations we went to for recruitment are engaged, in the main, in family support services including support for women. These are services that engages people from across the community. Youth clubs offer us a younger group and one that is attending this place for a very different (social) function to attendance at the mosque or support group. We explained in the article the decision to run four focus groups and to divide by gender and age. We also noted the possibilities for cross-group discussion, including challenge, that we sacrificed in making this choice.* *We did not specify exact age – just grouped people into younger or older categories  - the rationale, which we describe in the article,  was that the younger group are in a cohort likely to be deciding about their own marriage, the older group were made up of people who married at a time when high levels of parental input would have been commonplace. The division by gender was made because of long experience of working in this community and the preference, most marked in older groups, to discuss in same gender groups. Our sample was obtained by one of our authors (SI) attending our target sites and inviting people at events there to read an information sheet about the study and then, after some time for reflection and for questions,  to complete a consent form (see material provided in our Extended Data). We think this would be described as an opportunistic structured sample.  The only criteria for inclusion was that the person was from our target group, Bradford’s Pakistani heritage population, and that they consented to join the study. The decision to carry out 4 focus groups was a pragmatic decision shaped by research staff availability. Had we done more groups we would have expanded the data we would have been able to draw on. But we felt that four did give us rich insights into the questions the focus groups sought to address.* *We have added more detail to this section of the paper to better explain recruitment and sampling.*   Focus groups were conducted in English, potentially excluding less fluent participants and limiting the diversity of perspectives. This linguistic barrier should be acknowledged, and efforts to mitigate its impact should be described.

*We agree that the possibility of a linguistic barrier should be acknowledged and have added a note to this effect. It is possible that people either declined to be involved in the groups or contributed less than then might have wished to because of a lack of confidence in speaking in English in a group setting. We did try mitigating this risk by having focus group facilitators fluent in the languages most commonly used in the Bradford Pakistani community and offering participants the chance to speak in the language they felt most comfortable with. With have added a note to acknowledge and respond to the point that some people may have been excluded because of lack of confidence in speaking English. It is also possible that even if people agreed to join groups being undertaken in English that they felt inhibited in contributing because they thought their English was not good enough. Our experienced facilitators would have been alert to this, but we cannot be sure that nobody was put off contributing. * The authors mention Braun and Clarke's analysis framework, but the manuscript lacks key details, e.g., coding processes, validation methods (for instance, inter-coder reliability), and an audit trail for theme emergence. The use of subjective saliency criteria for prioritizing themes needs more justification. Including the focus group guide or discussion prompts as supplementary material would improve transparency and replicability.

*The methods section has now been revised to provide a more detailed explanation of the coding and analysis process. We did provide the focus group guide and its discussion prompts alongside copies of the information sheet we gave possible focus group recruits. These were in our Extended Data paper available in a link from the original version of our article. Specifically, in the Extended Data we provided a link to included :* *Focus Group Information Sheet/ Consent Form/ Topic Guide/questions (also a completed Standards for Reporting Qualitative Research (SRQR) form.)* *We accept the point about the subjectivity of using saliency criteria and we have added a little more about the process we undertook and about the background and experience of our data analysts – specifically their cultural and community knowledge which we think gives more confidence in the subjective aspect of this approach.* *In our analysis each data item received equal attention during the coding process, and themes were developed through a thorough and inclusive analysis of the dataset, rather than relying on isolated examples. We think this ensures the findings accurately represent the diversity of participant experiences and perspectives. In the context of our four focus groups, we felt we had sufficiently explored our research questions. * The introduction emphasizes the importance of consanguinity but relies too much on past descriptive statistics. A clearer research objective would enhance focus. It also fails to engage with broader theories, e.g., acculturation or health literacy models. Claims linking the decline in consanguinity to education and immigration policies are speculative and need stronger evidence. *We are aware that we may not have been clear enough about the hybrid nature of this paper. It is a companion piece to two papers,  Small et al (2024a ) on changing rates of consanguinity in the Born in Bradford study and on the changing characteristics of the consanguineous and Small et al.(2024b )  on the excess morbidity associated with consanguinity. We wanted to explore, using qualitative methods, the empirical findings from Small et al.(2024a ), specifically that there had been a considerable reduction in consanguinity over a nine year period in two demographically similar populations of Pakistani heritage in the city. The reduction was most marked in younger women, those educated to A Level or higher, and in those born in the UK or who moved to the UK when young. Our questions were designed to see if our respondents’ own beliefs were that consanguinity was reducing and that it was reducing mostly in the groups we identified. If they did recognise this what did they attribute it to and what significance did they think the changes might have? In relation to the second empirical study,  we wanted to see if the greater morbidity we attributed to consanguinity in Small et al. (2024b) was recognised and understood by our participants and to seek their input as to how best to discuss genetic risk with them and their community. Consequent upon these aims we constructed a focus group guide for our group conductors that sought to explore these pre-determined concerns. We included the detailed guide that focus group facilitators used in the Extended Data we submitted with our original paper. Although we did encourage participants to freely express their views there was also, in the focus group guide, a wish  to validate, expand on or problematise those themes we had seen elsewhere (ie in our empirical papers).*

*Our statistics are based on a number of empirical studies published since 2013 and the population we use to explore changes in rates of consanguinity was recruited up to 2019 and continues to be followed up and reported on. We don’t think this body of work can be called “past descriptive statistics”.* *Our research objective reflects a wish to report the community’s lived experiences and interpretations. These are  perspectives on consanguinity that are largely absent from a topic dominated by numerical data and policy discussions. In this context of  limited space for lived-experience discourse, our study offers a rich, thick description of the perspectives of a subset of the Pakistani heritage community in Bradford. This may serve as a basis for others to assess relevance or transferability to their own contexts. The methods section clearly delineates the study's focus, and the limitations section acknowledges that these findings are specific to this community and context.* *We do discuss broader theories in our section “Some thoughts on theoretical issues”. We do agree that a deeper examination of acculturation would add depth to our discussion of the impact of staying in education and expand on those sections that link being brought up in England or being in a particular age cohort with changing marriage patterns. But we do consider how far changing marital choices, and attitudes to consanguinity, are part of a culture change in which younger people seek to challenge older beliefs. We also ask  if they are manifestations of a wish to preserve community strengths that the younger people think can be achieved without cousin-marriage. Is this acculturation of within-group adaptation – we discuss this in our “Some thoughts…” section.    . But we accept we could have linked this more with the wide literature on this subject.  We have now added a section looking at how acculturation has been conceptualised and have put this alongside the description of hybrid societies that is already in our text. Likewise on health literacy models – we accept the reviewers point that exploring these could have been informative – we do tacitly talk about health literacy but we recognise that this should  be developed more. We have added a new short section including references to this literature and in particular have considered how  individual and community and social health literacy relate to our data.*

**.  

 The study captures diverse perspectives, but its conclusions are occasionally overgeneralized. Anecdotal health risks are discussed without considering other confounding factors and claims about declining consanguinity due to education and policy changes require more cautious framing and empirical support. *The health risk we base the discussion on is not anecdotal. The work begun in Bradford by Sheridan et al in 2013 identified consanguinity as the risk factor for increase in congenital anomalies. This finding has been widely commented on and, with some caveats (see Mobarak et al 2019), has been accepted by the academic community. Other confounding factors, including deprivation, did not make a difference in explaining the pattern of congenital anomalies reported in the Born in Bradford study.  We also think that claims about declining consanguinity in Bradford being linked to age of mothers, being born in the UK or moving at a young age and being educated to A level and higher are supported empirically (Small et al.2024b). Policy changes are more difficult to link – a potentially key policy change, a shift in Immigration law, was a little too late to be fully reflected in our findings – we do make this point in our text.  We wish to emphasise that our findings relate to the participants in Born in Bradford and conclusions we draw may not be transferrable to other communities of Pakistani heritage in the UK or elsewhere. Although we have said that in our text we have added a little more detail to underline the limited claims we seek to make. * Long, repetitive quotes weaken the findings. Condensed excerpts, clearer theme synthesis, and visual aids like thematic maps could enhance clarity and engage readers more effectively. Can the authors provide an account for this?** *We have now revised the quotes in the Findings section to remove some quotes and shorten others.  The categorisation of themes and sub themes is presented in Table 1 which is similar to a thematic map. We have now added more details by introducing a third column titled “theme components” for greater clarity.  We have sought to be clearer in our presenting  theme synthesis.*

  The authors might consider posting raw data, like excerpts or summaries, for transparency and meta-analysis. *We agree that posting raw data would enhance the transparency of our fundings and very-much support efforts to provide qualitative data in a form that allows meta-analysis. The Born in Bradford study this paper draws on has provided access to a considerable amount of its quantitative data but, not so far, qualitative data.  We are seeking to develop an on-line data repository and also make our data available via other qualitative repositories. We will adapt our consent procedures when such a resource is available. We recognise not doing this is a shortcoming of our study and have noted that in a revised section on “limitations”.*

 Overall, the study tried to tackle a sensitive topic using a qualitative approach, but methodological flaws, limited theoretical engagement, and speculative interpretations weaken its scientific rigour. Addressing these issues could improve its transparency, validity, and impact. *We hope we have responded to the reviewers’ concerns, in so far that its transparency and validity have been improved in the revised version. We have added sections on the areas the reviewer suggested additional literature to consider. We  have cut and reorganised our presentation of quotes from focus groups to reduce repetition. We think these changes/ additions strengthen the paper and we are grateful for the reviewer’s guidance.*
